# Successful implementation of technology in the management of Parkinson's disease: Barriers and facilitators

**DOI:** 10.1016/j.prdoa.2023.100188

**Published:** 2023-02-16

**Authors:** Arjonne Laar, Ana Ligia Silva de Lima, Bart R. Maas, Bastiaan R. Bloem, Nienke M. de Vries

**Affiliations:** a Department of Neurology, Radboud University Medical Center, Donders Institute for Brain, Cognition and Behavior, Reinier Postlaan 4, 6525 GC Nijmegen, the Netherlands; b Center of Expertise for Parkinson & Movement Disorders, Nijmegen, Reinier Postlaan 4, 6525 GC Nijmegen, the Netherlands

**Keywords:** Parkinson’s disease, Technology, Barriers, Facilitators, Literature review, telemedicine

## Abstract

**Background:**

Parkinson’s disease (PD) is a progressive neurodegenerative disease with a fast increasing prevalence. Several pharmacological and non-pharmacological interventions are available to alleviate symptoms. Technology can be used to improve the efficiency, accessibility and feasibility of these treatments. Although many technologies are available, only few are actually implemented in daily clinical practice.

**Aim:**

Here, we study the barriers and facilitators, as experienced by patients, caregivers and/or healthcare providers, to successful implement technology for PD management.

**Methods:**

We performed a systematic literature search in the PubMed and Embase databases until June 2022. Two independent raters screened the titles, abstracts and full texts on: 1) people with PD; 2) using technology for disease management; 3) qualitative research methods providing patients’, caregivers and/or healthcare providers’ perspective, and; 4) full text available in English or Dutch. Case studies, reviews and conference abstracts were excluded.

**Results:**

We found 5420 unique articles of which 34 were included in this study. Five categories were made: cueing (n = 3), exergaming (n = 3), remote monitoring using wearable sensors (n = 10), telerehabilitation (n = 8) and remote consultation (n = 10). The main barriers reported across categories were unfamiliarity with technology, high costs, technical issues and (motor) symptoms hampering the use of some technologies. Facilitators included good usability, experiencing beneficial effects and feeling safe whilst using the technology.

**Conclusion:**

Although only few articles presented a qualitative evaluation of technologies, we found some important barriers and facilitators that may help to bridge the gap between the fast developing technological world and actual implementation in day-to-day living with PD.

## Highlights

• Technologies are available but not yet widely implemented for PD management.
• Barriers for using technology include unfamiliarity, costs and technical issues.
• Facilitators are good usability, beneficial effects and feeling safe using technology.
• Technology-specific barriers and facilitators should be taken into account.
• Users should be involved in developing technologies for successful implementation.

## 1 Introduction

Parkinson’s disease (PD) is a progressive neurodegenerative disease with a worldwide prevalence of 6.1 million people in 2016 [1]. This prevalence has increased by 145 % since 1990 [1] and is expected to increase even further in the coming decades [2]. PD is characterized in part by motor symptoms, including tremor at rest, rigidity, bradykinesia and postural instability [3]. In addition, non-motor symptoms such as cognitive problems, sleep disorders, autonomic dysfunction and sensory problems are also commonly experienced [4]. Both pharmacological [5] and non-pharmacological [6] therapies can be used to manage the disease. So far, however, there is no disease-modifying treatment available [7]. Moreover, PD management is complex for a number of reasons, including the lack of objective outcome measures to personalize treatments [8], the occurrence of intermittent or fluctuating motor symptoms [9] and the long travel distance to specialized healthcare professionals [2].

Successful implementation of technology may improve disease management and thereby the quality of life of people with PD [10]. Examples include remote monitoring to continuously monitor symptoms of people with PD at home [11]. This is expected to add valuable information to the snapshots of subjectively gathered experiences during a normal consultation, making treatment more personalized. In addition, remote consultation can be used to reach people with PD living in remote areas [12]. As a result, quality of received care may no longer depend on the location where someone lives.

Technology can also be used to deliver interventions remotely. One important area relates to mobility. Loss of independence has a major impact on the quality of life in people with PD [13]. Technology can be used to improve daily life functioning, for example by using cueing interventions to improve walking capacity [14] or forms of remote therapy to improve balance and postural stability [15].

In the last few years, many technological interventions have been developed and piloted [16]. However, actual implementation of these technologies is mostly lacking. This is caused by several aspects, including small sample sizes of subjects in most studies, which reduces the generalizability of the technology [16]. The aim of this review is 1) to provide an overview of the barriers and facilitators for the technologies, as experienced by patients, caregivers and/or healthcare professionals, and 2) to give recommendations for further development of technologies. More insight into the barriers and facilitators may give direction to the future implementation of technology for PD management.

## 2 Methods

We performed a systematic literature search in the online databases PubMed and Embase. The search aimed at identifying studies listing barriers and facilitators of technologies used to improve the disease management in people with PD. We used the following search terms with their synonyms in title/abstract and as MeSH (PubMed) or Emtree (Embase): “requirements” or “facilitators” or “barriers” or “feasibility” or “usability” or “implementation” in combination with “technology” or “telemedicine” or “sensors” and “Parkinson”. In addition to the search in the online databases, we reviewed the reference lists of included articles. The final search strategy can be found in Appendix I.

**Fig. 1:**
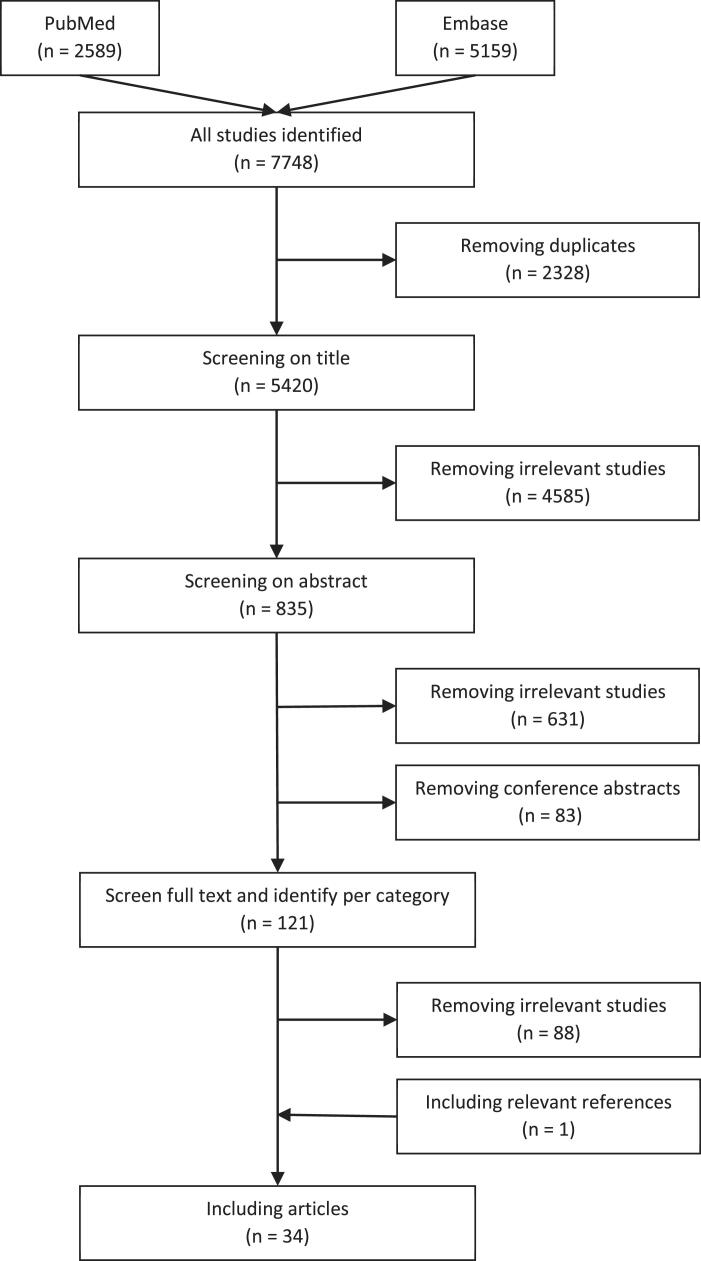
Flowchart of the literature search strategy.

Articles were included when they met the following inclusion criteria: 1) focusing on people with PD; 2) using technology for disease management; 3) using a qualitative methodology focusing on the perspective of the patient, caregiver and/or healthcare professional, and; 4) full text available in English or Dutch. Case studies, reviews and conference abstracts were excluded (Fig. 1).

We searched the literature until June 2022 and this resulted in a total of 7748 articles. After excluding all duplicates, two independent raters (AL and ALSL) screened the titles and abstracts of the remaining 5420 studies. Subsequently, they screened the full text of the selected 121 articles. Disagreement was solved by a third independent rater (NMdV).

Data was extracted from the included articles using a predefined table. The extracted variables included: author, year of publication, included population, intervention used, methods used for qualitative data collection, and barriers and facilitators found. Based on the interventions that were described, articles were subdivided in the following five categories: cueing, exergaming, remote monitoring using wearable sensors, telerehabilitation and remote consultation.

## 3 Results and recommendations

We included a total number of 34 relevant articles. Table 1 provides an overview of the participant characteristics, interventions, methods and findings of these 34 articles included in this review [17], [18], [19], [20], [21], [22], [23], [24], [25], [26], [27], [28], [29], [30], [31], [32], [33], [34], [35], [36], [37], [38], [39], [40], [41], [42], [43], [44], [45], [46], [47], [48], [49], [50]. Of these studies, three focused on a cueing intervention [17], [18], [19], three focused on exergaming [20], [21], [22], ten focused on remote monitoring using wearable sensors [23], [24], [25], [26], [27], [28], [29], [30], [31], [32], eight focused on telerehabilitation [33], [34], [35], [36], [37], [38], [39], [40] and ten focused on remote consultation [41], [42], [43], [44], [45], [46], [47], [48], [49], [50]. The barriers we found across categories included the unfamiliarity with the technology itself, high costs, technical issues and (motor) symptoms hampering the usability of some technologies. General facilitators for the implementation of technologies were good usability, experiencing beneficial effects of using the technology and feeling safe whilst using the technology. In the next paragraphs we will discuss the barriers and facilitators for each category separately, and give recommendations for further development based on the experiences of patients and/or healthcare providers. Detailed results per category can be found in Table 2, Table 3, Table 4, Table 5, Table 6.

**Table 1:** Overview of articles on the barriers and facilitators for implementation of technology for persons with PD.

**Category: cueing**					
**Study**	**Population**	**Intervention**	**Design**	**Barriers**	**Facilitators**
Amini et al. (2019)	15 persons with PD (80.0% male); Mean age: 72.06 years (SD not reported); Mean time since diagnosis: 8.66 years (SD not reported).	Detection of freezing of gait and delivery of cueing (laser lines) with a RGB-D camera.	3 focus groups (n=5 persons with PD per group)	- Not portable.	- Easy to use; - Accurate; - Beneficial (improves mobility and walking performance); - No privacy concerns.
Janssen et al. (2017)	25 persons with PD (76% male); Median age: 72 (range: 65-79) years; Median time since diagnosis: 11 (range: 3-20) years.	Comparing types of 3D augmented visual cues delivered by smart glasses to: 1) conventional 3D cueing on the floor, 2) and auditory cueing via a metronome and 3) no cueing.	Structured semi-open interviews	Barriers to use of technology: - Low usability; - Low willingness to use smart glasses in everyday life; - Uncomfortable; - Aesthetics; - Diminished field of view; - Heavy; - Big size. Barriers to augmented visual cues: - Distracting; - Blockage of visual field; - Unfamiliar with usage of smart glasses.	- No additional effort required for using the smart glasses; - Walking with smart glasses is easy to learn.
Zhao et al. (2016)	12 persons with PD (75% male); Mean age: 66.8 ± 6.8 years; Mean time since diagnosis: 13.6 ± 6.7 years.	Smart glasses as a new technology to deliver external cueing (metronome, flashing light or optic flow).	Structured semi-open interviews	Barriers to use of technology: - Difficult to synchronize to the optic flow; - Difficult to walk while focusing on optic flow cues; - Placement of the visual display in the upper right corner. Barriers to optic flow: - Annoying; - Distracting; - Demanding too much concentration; - Hard to see.	- Willing to use smart glasses at home; - Cues were delivered at comfortable speed; - Smart glasses were easy to use; - Instructions presented on the screen were clear to read; - Bone-conducting headphone was appreciated because the metronome was less audible to others around them.

**Category: exergaming**					
**Study**	**Population**	**Intervention**	**Design**	**Barriers**	**Facilitators**
Galna et al. (2014)	Part 1: 2 persons with PD H&Y: 50% 2, 50% 3;1 caregiver (additional characteristics not reported).Part 2:9 persons with PD (66.7% male); Mean age: 68.2 ± 8.3 years; H&Y: 33.3% 1, 55.6% 2, 11.1% 3.	A rehabilitation game aiming at training dynamic postural control using a RGB-D camera.	Part 1: design workshopPart 2: semi-structured interviews	Part 1: - Thematic of the game: adventure or complex narrative based game (especially science fiction); - Combination of puzzles with physical tasks overly complicated; - Pace of the game too fast. Part 2: - Visuals (inability to distinguish different objects); - Difficulty with stepping task; - More challenging for cognition than for balance; - Game not suitable with hypotension; - Implementation in daily life depends on the price.	Part 1: - Solo play; - ‘Real life’ events instead of fantasy elements; - Cartoon style graphics instead of realistic renderings; - Puzzles; - Outdoor scenarios; - Able to identify with a cartoon avatar which mirrored their actions; - Satisfying sound effects associated with actions. Part 2: - Enjoyment of playing the game; - Feeling safe whilst playing the game; - Participants could imagine playing the game at home; - Participants would enjoy playing the game with others; - Competition as important gameplay factor.
Natbony et al. (2013)	16 persons with PD (68.8% male); Mean age: 63.1 ± 9.8 years; Mean H&Y: 2.	An interactive dance videogame “Dance Dance Revolution” that incorporates cognitive movement strategies, physical capacity, balance training and cueing.	Focus groups	- Distracting or confusing interface; - Financial cost; - Limited range of movements; - Cannot customize game display; - Increased change of falling; - Music available not appreciated.	- Fun; - Easy to use; - Improves balance or coordination; - Challenging; - Full-body aerobic activity.
Sanchez-Herrera-Baeza et al. (2020)	6 persons with PD (83.3% male); Mean age: 74.50 ± 4.72 years; H&Y: 33.3% 2, 66.6% 3.	Serious gaming for upper limb mobility via virtual reality technology (Oculus Rift 2 plus leap motion controller – OR2-LMC).	Semi-structured in-depth interviews	- Fatigue; - Short interval between rounds in the game; - Monotony of video game activities; - Fear of new challenges and activities; - PD tremors interfering with the ability to perform the task.	- Sense of competition against the machine; - Improvement of personal scores; - Overcoming challenges makes patient feel closer to family and more competent in daily living activities; - Helps identifying limits while striving to overcome these; - More awareness; - Increases patient empowerment; - Increases support among persons with PD.
**Category: remote monitoring using wearable sensors**					
**Study**	**Population**	**Intervention**	**Design**	**Barriers**	**Facilitators**
AlMahadin et al. (2020)	Part 1: 3 healthcare professionals and 1 Parkinson local supporter (25.0% male); Mean age: 57.75 ± 6.29 years. Part 2:12 persons with PD (58.3% male); Mean age: 73.83 ± 10.69 years (range: 56-88 years); Mean time since diagnosis: 8.5 ± 7.29 years (range: 2-24 years).	Wearable technology system for the diagnosis and assessment of motor symptoms in Parkinson’s disease.	Part 1: semi-structured interviews Part 2: focus groups	Part 1: - Device visibility in early-disease staged persons with PD; - Expensive; - Difficult to interpret the data and results. Part 2: - Device placed on an undesired part of body (e.g. neck or ankle); - Fear or dislike of modern technologies.	Part 1: - Comfortable; - Easy to use; - Non-invasive; - Easily worn under clothes without catching/snagging; - Water-resistant; - Washable; - Durable; - Easy to fasten; - Less traveling to the hospital for persons with PD; - Additional functionalities (e.g. fall detection, medication reminders). Part 2: - Comfortable - Non-invasive; - Wrist-worn device; - Waterproof; - Durable; - Small; - Easy to fasten; - Identification by the device as persons affected by PD; - No privacy concerns; - Additional functionalities (e.g. medication reminders, help call button).
Cancela et al. (2013)	24 persons with PD (75.0% male); Age (range): 52 – 76 years (additional characteristics not reported).	Wearable sensors to detect and quantify symptoms related to Parkinson’s disease.	Interviews	- Discomfort/pain; - Visibility of the device; - Technical issues; - Design problems (e.g. too small text).	- No perception of harm; - No obstruction of daily activities by the device; - Acceptance of touch-screen PC.
Cancela et al. (2014)Wearability	32 persons with PD (68.8% male; additional characteristics not reported).	Wearable sensors for remote monitoring of persons with PD.	Questionnaire and interviews	- Visibility of the device; - Help needed for attachment; - Not waterproof; - Allergic reactions, excess heat and sweat caused by the device; - Attachment was too tight (too short straps).	- High wearability; - No obstruction of daily activities by the device.
Cancela et al. (2014) Feasibility	11 persons with PD (63.6% male); Mean age: 65.5 ± 8.2 years (additional characteristics not reported).	Wearable sensors to detect and quantify symptoms related to Parkinson’s disease.	Interviews	- Discomfort/pain; - Visibility of the device; - Attachment was too tight (too short straps).	- No perception of harm; - No obstruction of daily activities by the device.
Elm et al. (2019)	14 clinicians (100% movement disorder neurologists; additional characteristics not reported).	The Fox Wearable Companion App and a wearable sensor for remote monitoring of Parkinson’s Disease symptoms.	Focus groups	- Interface listing results of monitoring difficult to interpret.	- Monitoring medication compliance; - Monitoring activity level and night-time activity; - Hourly displays or daily displays for the night-time activity component.
Fisher et al. (2016)	34 persons with PD; Mean age: 69 (range: 50 – 86) years; Mean time since diagnosis: 10 (range: 2 – 26) years.	Wrist-worn sensor to assist in treatment decisions and evaluation of new treatments.	Questionnaire	- Device too large; - Attachment too loose; - Uncomfortable and soggy when wet; - Awkward for persons with tremor; - Pin came out of the device; - Difficult fasten the strap of the device during OFF; - Velcro of the device not comfortable.	- No problems wearing it when it has a watch face or when it can be covered by sleeves; - No interference with daily life; - Comfortable.
Hermanns et al. (2019)	5 persons with PD (60.0% male); Mean age: 73.00 ± 4.95 years; Mean time since diagnosis: 6.0 ± 2.99 years.	Wearable sensor for monitoring physical activity of persons with PD, in combination with an iPad to view exercise videos and access the online support group.	Questionnaire	- Difficulties due to unfamiliarity with iPad; - Unable to wear the sensor due to skin sensitivity.	- Clear and helpful videos; - Using the sensor was beneficial for the health of persons with PD.
Memedi et al. (2011)	14 neurologists (additional characteristics not reported); 7 nurses (0% male; Mean age 49 (range: 38-61) years.	Web application and a wearable device (hand computer with touchscreen) for monitoring persons with PD and assisting decision making concerning treatments.	Questionnaire	- Not reported.	Neurologists: - Web application easy to understand; - Web application easy to use; - Ability to identify persons with PD who are not doing well; - Facilitated follow-up optimization of an individual’s treatment. Nurses: - Very useful; - Results showed agreement with qualitative observations; - Comparisons between persons with PD are possible.
Timotijevic et al. (2020)	47 clinicians (44.7% consultant neurologists, 6.4% Parkinson’s disease nurses, 19.1% general practitioners, 8.5% physiotherapists, 6.4% occupational therapists, 10.6% psychologists, 4.3% speech therapists; additional characteristics not reported).	mHealth Clinical Decision Support Systems using easily portable devices such as smart phones, wristbands and sensor insoles to capture objective data of persons with PD about their fluctuating condition.	Hierarchical Task Analysis	- Not reported	- Useful since most clinicians had little or no access to continuous objective data on PD motor symptoms.
Virbel-Fleischman et al. (2022)	22 persons with PD (40.9% male); Median age: 65.5 (range: 41 – 79) years; Median disease duration: 7.5 (range: 1 – 17) years; 9 healthcare professionals (66.7% neurologist, 33.3% nurses specializing in PD; 33.3% male).	Body-worn sensors for monitoring of motor symptoms.	Semi-structured interviews	Persons with PD: - Concerns about using it properly; - Visibility of the sensor; - Concerns about privacy. Healthcare professionals: - Replacement of patient-doctor relationship; - Need of helping patients with the device; - Logistical support (i.e. how to set up the monitoring, how to give/return the device etc).	Persons with PD: - Ease of use; - More objective insight in symptoms by healthcare professionals. Healthcare professionals: - Beneficial for adjustment in treatment; - Helps to minimize or avoid visits or hospitalization.

**Category: telerehabilitation**					
**Study**	**Population**	**Intervention**	**Design**	**Barriers**	**Facilitators**
Flynn et al. (2022)	17 persons with PD - 9 in home based group (77.8% male); Age (range): 50 – 89 years; Mean time since diagnosis: 4.1 ± 3.9 years;H&Y: 33.3% 1, 44.4% 2, 22.2% 3. - 8 in center based group (87.5% male); Age (range): 50 – 89 years;Mean time since diagnosis: 7.4 ± 4.9 years;H&Y: 25.0% 2, 75.0% 3.	Predominately home-based exercise in comparison to predominately center-based exercise.	Semi-structured interviews	Home-based compared to center-based: - Challenging and at times frustrating due to PD symptoms; - Challenging due to not having the same equipment and space as at the center; - More need for support and feedback; - Difficult to maintain motivation to exercise at home; - Isolating to be exercising at home instead of at the center;	Home-based compared to center-based: - Exercise was prescribed by a health professional with consideration given to individual needs, targeted to their PD and progressed; - Experiencing improvements in both motor and non-motor impairments; - Sense of achievement and satisfaction after completion the exercise; - Clear description of exercises in app or on paper; - First exercises in the center gave confidence and knowledge to exercise at home; - The flexibility to complete the exercise at a time which was convenient; - No travel requirements.
Kwok et al. (2022)	8 persons with PD (50% male); Mean age: 63.1 ± 5.4 years; H&Y: 100% 3.	mHealth-delivered home-based mindfulness yoga program.	Semi-structured interviews	- Some concerns about the execution of yoga postures.	- Interactive and interesting program; - Convenience; - No travel issues; - No adverse weather; - No health measures against COVID-19; - No safety concerns related to social movement activities in the local community; - Not much additional equipment needed (only a yoga mat, chair and one smart device installed with Zoom); - Experiencing the benefits of yoga (e.g. calmness, balance, mobility and gait).
Lai et al. (2020)	20 persons with PD - 10 in TAE group (70.0% male); Mean age: 63.4 ± 10.4;Mean time since diagnosis: 6.55 ± 4.52 years;Mean H&Y: 2.15 ± 0.47. - 10 in SRE group (70.0% male); Mean age: 70.8 ± 7.1;Mean time since diagnosis: 7.55 ± 4.78 years;Mean H&Y: 2.3 ± 0.63.	Telecoach-assisted exercise (TAE) in comparison to self-regulated exercise (SRE) groups.	Semi-structured interviews	TAE: - Technical issues (e.g. internet instability); - Using technology needed a learning curve. SRE: - Technical issues.	TAE: - Convenience; - Monitoring capability of the telehealth system; - Support increases motivation; - Personalization increases confidence to exercise and/or using technology. SRE: - Potential benefits; - Monitoring capability of the telehealth system; - Convenience; - Increases accountability.
Morris et al. (2021)	12 persons with PD (25.0% male); Median age: 59.5 years (IQR: 58.0 – 63.0); H&Y: 33.3% 1, 41.7% 2, 8.3% 2.5, 16.7% 3.	The online delivery of the therapeutic dancing classes ‘ParkinDANCE’.	Semi-structured interviews	- Need for stable internet services, access to digital devices, IT literacy and access to technical support.	- Beneficial effects (e.g. improved self-confidence and more able to venture into public spaces); - Physically and mentally challenging, but still appropriately tailored to capability of persons with PD; - Variety in dance genres but progression in complexity; - Variety of music, but a clear beat was key; - Input in music choice because of individual preferences for music; - Instructor qualities (e.g. perception of dance proficiency, teaching skills and the ability to communicate positively, with respect, empathy, patience and understanding, and making the dance sessions fun and challenging); - Instructor with knowledge about movement disorders and PD; - Safety checks before and during each session.
Roswell et al. (2022)	37 persons with PD (54% male); Mean age: 71 (range: 57 – 84) years; Median time since diagnosis: 10 (range: 1.5 – 25) years; H&Y: 11% 1, 27% 2, 57% 3, 5% 4.	Individually tailored, progressive home-based exercise and strategies to avoid falls.	Semi-structured interviews	- Time consuming; - The decline in visits by the physiotherapists as the programme progressed; - Boredom; - Technical issues; - Feeling limited because the exercises and strategies were not accessible on a portable device; - The use of equipment to increase the intensity of exercises; - PD-related symptoms made it difficult to participate (e.g. due to OFF or poor memory); - Feeling embarrassed or self-conscious about exercise.	- Greater effort and commitment in knowing that the physiotherapist would be returning; - Support and encouragement from one’s partner, spouse or carer; - Written material about the programme; - Possibility to employ the strategies into daily routines.
Stack et al. (2016)	5 persons with PD (40.0% male); Mean age: 74.2 ± 3.3 years; Mean time since diagnosis: 8.8 ± 3.2 years; H&Y: 60.0% 4, 40.0% 3.	A combination of a RGB-D camera and wearable sensors to record in-home falls and movements.	Observational study	- Feeling of being watched; - Sending alerts to caregivers (resulting in caregivers hurrying back when they are out, although that is rarely what the faller needed or wanted); - Persons with PD unstable for necessary tasks for the application of the wearables.	- Not cumbersome; - No hinder or distraction for participants; - Comfortable way of applying the sensors.
Torriani-Pasin et al. (2022)	19 persons with PD (57.9% male); Mean age: 69.80 ± 10.12 years; Mean time since diagnosis: 7.0 ± 4.13 years; H&Y: 10.5% 1, 15.8% 1.5, 51.8% 2, 36.8% 2.5, 21.0% 3.	A telemonitoring‐based physical exercise program.	Questionnaire	- Telemonitoring issues (e.g. internet connectivity, technology, issues related to the program); - Lack of safe space to exercise; - Need to have a companion for safety reasons; - Fear of injury; - PD-related issues (e.g. freezing of gait, tremor, lack of motor skills and dual-task performance difficulty).	- Not reported.
Walton et al. (2022)	23 persons with PD (26% male); Mean age: 70.4 ± 7.4 years; Mean time since diagnosis: 8.0 ± 6.4 years.	Digital dance class.	Questionnaire and focus groups	- Technical issues (e.g. unsynchronized sound, limited screen size); - Cognitively challenging leading to mental tiredness; - Lack of social contact; - Space limitations (i.e. feeling inhibited from making larger movements).	- Ease of access; - Lack of preparation needed; - Enjoyable; - Adaptation for people with PD and one’s individual abilities; - Experiencing physical changes (e.g. better balance, less rigidity and a longer stride length); - Appreciated music.

**Category: remote consultation**					
**Study**	**Population**	**Intervention**	**Design**	**Barriers**	**Facilitators**
Anghelescu et al. (2022)	22 persons with PD (72.7% male); Mean age: 70.5 (range: 51 – 79) years (additional characteristics not reported).	Virtual care as result of COVID-19.	Virtual, in-depth semi-structured interviews	- Simultaneous speaking making it difficult to converse; - Less personal connection; - Increased difficulty conveying notes or updates virtually.	- Convenience; - Efficiency; - Saving the hassle of driving and parking.
Dorsey et al. (2010)	14 persons with PD (50.0% male); Mean age: 71.4 years (additional characteristics not reported).	Remote care in comparison with usual care.	Focus groups	- Technical issues (e.g. dropped signals, trouble hearing the doctor); - Challenges in changing doctors (previous physicians not receptive to this change); - Travel to the telemedicine site; - Mixed feelings about completing telemedicine visits at home without medical and technical support.	- Decreased travel burden; - Access to high quality, dedicated PD experts; - No feelings of rush; - Feeling that doctor listens to concerns; - Convenient; - Easy communication.
Evans et al. (2020)	61 persons with PD (72.1% male); Mean age: 70.1 years (range: 50-86 years); Mean bradykinesia score: 33.7; Mean levodopa equivalent dose: 463.98 mg.	A virtual clinic which combines phone consultations and reports from the Parkinson’s KinetiGraph (a wrist-worn device).	Questionnaire	- Not comfortable since patient never met the doctor before; - Difficulty speaking on the phone; - Phone call more stressful than face to face contact; - Lack of insight in body language; - No personal touch.	- Decreased travel burden; - Time saving; - Useful as addition to face to face contact; - Better preparation of the appointment by patient.
Mammen et al. (2018)	195 persons with PD (53.3% male) Mean age: 66.4 ± 8.1 years; Mean PD duration: 8.0 ± 5.6 years; 20 physicians (Parkinson’s specialists; additional characteristics not reported).	Usual care in comparison with usual care augmented by four virtual visits with a PD specialist delivered directly into the home.	Online survey with open-ended questions	Lack of personal benefits of the virtual house call: - Inconvenient (technical problems); - Frustrating; - No benefit versus usual care (e.g. when living close to physician or already satisfactorily engaged with a PD specialist); - Lack of continuity of care. Decreased quality of care: - Technical troubles substantially affected care (e.g. connection trouble, audio and visual difficulties); - Trouble conducting exam; - Less control over situation. Poorer quality of interpersonal engagement: - Difficult communication; - Less comfortable; - Lack of intimacy; - Lack of confidence in physician.	Personal benefits: - Less travel; - Less wait time; - Less stress; - Less expense; - More comfort; - More convenient; - Access to specialist. Perceived quality of care: - More time with doctor; - More frequent visits; - More thorough assessment; - Benefitting from care. Quality of interpersonal engagement: - Good communication; - Felt seen and understood; - Liked doctor/patient.
Peacock et al. (2020)	18 persons with PD completed the survey; 8 participants in the focus group (62.5% persons with PD; Mean age: 71.6 ± 7.5 years; 37.5% caretaker; Mean age: 69.7 ± 2.5 years).	Telehealth to improve access of a physician for persons with PD.	Survey and focus groups	- Concern that family physicians and general neurologists did not have the necessary expertise to manage their PD; - An insufficient number of neurologists in their communities; - Long wait times for initial consultations; - Shortage of expertise in smaller centers; - Lack of access to multidisciplinary care and advanced therapies (e.g. DBS); - Less comfortable for elderly; - Losing a personal connection with their neurologists.	- No travel costs; - No travel risks (winter appointments on poor road conditions); - Less strain on patient and caregiver; - Ability to be assessed at home; - Access to expertise.
Quinn et al. (2019)	8 persons with PD (75% male); Mean age: 68.5 ± 8.3 years; Mean time since diagnosis: 4.5 ± 1.5 years; Mean H&Y: 1.94 ± 0.68.	Delivering a group-based speech maintenance programme into the home environment via telerehabilitation to persons with PD.	Questionnaire	- Technical issues.	- Convenience; - Treatment effectiveness; - Opportunity to meet other persons with PD; - Appropriate frequency; - Time saving; - Effectivity.
Stillerova et al. (2016)	11 persons with PD (63.6% male); Median age: 69.0 years (IQR: 57.0–76.0); Median time since diagnosis: 3.0 years (IQR 2.5–9.5).	Remotely assessing the symptoms of PD via computers and webcams available at home.	Feedback forms	- Inability to see the participants’ entire bodies because of space constraints; - Technical issues; - Limits to technology; - Concern for others’ experiences.	- Convenience; - Efficiency; - Reduced traveling; - Ability to displaying symptoms that are intermittent; - Positive feeling on interaction via teleconference.
Tarolli et al. (2020)	38 persons with PD (71.1% male); Mean age: 64.3 ± 10.4 years; Mean time since diagnosis: 2.4 ± 0.9 years; H&Y: 57.9% 1, 42.1% 2.	Remote research visits in observational studies in PD.	Survey	- Technical issues; - Size of the tablet too small; - Remote assessment feels incomplete.	- High satisfaction; - Good connection; - Convenience; - Comfort; - Ease of scheduling; - Improved safety (not having to drive).
Wannheden et al. (2020)	7 persons with PD (42.6% male, age not reported); 9 healthcare professionals (44.4% neurologists, 33.3% nurses, 22.2% physiotherapists; additional characteristics not reported).	eHealth system to realize co-care between persons with PD and healthcare professionals.	Workshops	- No access to care for all persons with PD; - More teamwork of professionals, each having clarifying roles and responsibilities; - Additional administrative workload; - Overuse of opportunity to report health issues by persons with PD, increasing professionals’ workload.	- Electronic pre-visit form; - Patient self-tracking of health; - Graphical overview of health data; - Clinical decision support functionality; - Provision of self-care recommendations; - Text-based messaging for asynchronous communication.
Willows et al. (2020)	Neurologists and Duodopa nurse specialists (number and characteristics not reported).	Remote consultation using a video communication system for alternative titration procedures, allowing Levodopa-carbidopa intestinal gel initiation at home.	Questionnaire for neurologists and DNS	- Lack of reliable means for determining rigidity, physical examination and the pull-test for postural instability.	- Not reported.

**Table 2:** Overview of the barriers and facilitators of cueing.

**Category: cueing**			
**Barrier**	**Study**	**Facilitator**	**Study**
Low usability (e.g. not portable, uncomfortable, diminished field of view, big size of the smart glasses, difficult to synchronize to the cues, difficult to walk while focusing on the cues)	[17], [18], [19]	Usability (e.g. no additional effort required for using the smart glasses, walking with smart glasses easy to learn, instructions on the screen were clear to read)	[17], [18], [19]
Augmented visual cues not optimal	[18]	Accuracy	[17]
Unfamiliar with usage of smart glasses	[18]	Beneficial (e.g. improves mobility and walking performance)	[17]
Optic flow not optimal (e.g. annoying, distracting, demanding too much concentration, hard to see, blockage of visual field)	[18], [19]	No privacy concerns	[17]
Low willingness to use smart glasses in daily life	[18]	Acceptance	[19]
		Cues delivered at comfortable speed	[19]
		Bone-conducting headphone appreciated because the metronome is less audible to others around the one wearing it	[19]

**Table 3:** Overview of the barriers and facilitators of exergaming.

**Category: exergaming**			
**Barrier**	**Study**	**Facilitator**	**Study**
Features of the game (e.g. game pace too fast, distracting or confusing interface, monotony of the game)	[20], [21], [22]	Features of the game (e.g. puzzles, full body activity, improvement of personal score)	[20], [21], [22]
Difficulty with stepping tasks	[20]	Enjoyment of playing the game	[20], [21]
More challenging for cognition than for balance	[20]	Feeling safe whilst playing the game	[20]
Game not feasible for persons with hypotension	[20]	Participants could imagine playing the game at home	[20]
Financial costs	[20], [21]	Participants would enjoy playing the game with others	[20]
Increased chance of falling	[21]	Easy to use	[21]
Fatigue	[22]	Improves balance or coordination	[21]
Fear of new challenges and activities	[22]	Overcoming challenges makes patient feel closer to family and more competent in daily living activities	[22]
PD tremors interfering with the ability to perform the task	[22]	Helps identifying limits while striving to overcome these	[22]
		More awareness	[22]
		Helped participants to focus on their treatment and be more involved in it	[22]
		Supporting each other	[22]

**Table 4:** Overview of the barriers and facilitators of remote monitoring using wearable sensors.

Category: remote monitoring using wearable sensors			
Barrier	Study	Facilitator	Study
Suboptimal attachment of the device	[25], [26], [28]	No perception of harm	[23], [24], [26]
Device uncomfortable	[25], [28]	Neither obstructing daily activities by using the device nor preventing persons with PD from doing daily tasks	[24], [25], [26]
Interface suboptimal	[24], [28]	Acceptance of touch-screen PC	[24]
Discomfort of allergic reactions	[29], [24], [25], [26]	High wearability	[23], [25]
Visibility of the device	[32], [23], [24], [25], [26]	For clinicians:	
		- Monitoring medication compliance	[27]
		- Monitoring activity level and night-time activity	[27]
		- Hourly displays for the night-time activity component	[27]
		- Useful since most clinicians had little or no access to continuous objective data on PD motor symptoms	[31]
		- Beneficial for adjustment in treatment	[32]
		- Helps to minimize or avoid visits or hospitalization	[32]
Technical issues	[24]	For neurologists:	
		- Easy to understand	[30]
		- Easy to use	[30]
		- Ability to identify persons with PD who are not doing well	[30]
For clinicians:		For nurses:	
- Data and results difficult to interpret	[23], [27]	- Useful	[30]
- Replacement of patient-doctor relationship	[32]	- Results showed agreement with qualitative observations	[30]
- Need of helping patients with device	[32]	- Comparisons between persons with PD are possible	
- Logistical support (i.e. how to set up the monitoring, give/return the device etc)	[32]		[30]
Awkward for persons with tremor	[28]	Clear and helpful videos	[29]
Difficulties due to unfamiliarity with the technology	[29], [32]	Using the activity tracker beneficial for the health of persons with PD	[23]
Expensive	[23]	Less traveling to the hospital for the patient	[23]
Device on an undesired part of the body	[23]	Additional functionalities	[23]
Fear of dislike of modern technologies for elderly	[23]	No privacy concerns	[23]
Concerns about privacy	[32]	Easy to use	[23], [32]
		More objective insights in symptoms by their healthcare professionals	[32]

**Table 5:** Overview of the barriers and facilitators of telerehabilitation.

**Category: telerehabilitation**			
**Barrier**	**Study**	**Facilitator**	**Study**
Technical issues	[35], [36], [37], [39], [40]	Convenience	[34], [35]
Feeling of being watched	[38]	Monitoring capability of the telehealth system	[35]
Sending alerts to caregivers (resulting in caregivers hurrying back when they are out, although that is rarely what the faller needed or wanted)	[38]	Support increases motivation to exercise	[35], [37]
Instability of persons with PD hinders necessary tasks for calibration of the wearables	[38]	Personalized leading to confidence to exercise and/or using technology	[35]
Challenges in exercising at home due to PD symptoms	[33], [37]	Potential benefits of telehealth (e.g. exercising when and where you like, or possibility to employ strategies into daily routines, no additional equipment needed)	[33], [34], [35], [37]
Challenges in exercising at home due to not having the equipment and space at home	[33], [37], [39], [40]	Accountability	[35]
More need for support and feedback	[33]	Beneficial effects (e.g. improved self-confidence and more able to venture into public spaces, improvements in motor- and non-motor symptoms, sense of achievement and satisfaction)	[33], [34], [36], [40]
Difficult to maintain motivation	[33]	Physically and mentally challenging, but still appropriately tailored to capability of persons with PD	[36]
Isolating / lack of social contact	[33], [40]	Variety in dance genres but progression in complexity	[36]
Concerns about the correct execution of exercises / postures	[33], [34]	Variety of music, but a clear beat was key	[36], [40]
Boredom	[37]	Input in music choice because of individual preferences for music	[36]
Time consuming	[37]	Instructor qualities (e.g. perception of dance proficiency, teaching skills and the ability to communicate positively, with respect, empathy, patience and understanding, and making sessions fun and challenging)	[33], [36]
Feeling limited because the exercises and strategies were not accessible on a portable device	[37]	Instructor with knowledge about movement disorders and PD	[33], [36]
Feeling embarrassed or self-conscious about exercise	[37]	Safety checks before and during each session	[36]
The decline in visits by the physiotherapists as the program progressed	[37]	Not cumbersome	[38]
Need to have a companion for safety reasons	[39]	No hinder or distraction for participants	[38]
Fear of injury	[39]	Comfortable way of applying the sensors	[38]
PD-related issues (e.g. freezing of gait, tremor, lack of motor skills and dual-task performance difficulty)	[39]	Related to traveling (no requirements, no issues, no adverse weather to face)	[33], [34]
Cognitively challenging leading to mental tiredness	[40]	Clear description of the exercises	[33], [37]
		First exercises in the center gave confidence and knowledge to exercise at home	[33]
		No health measures against COVID-19	[34]
		No safety concerns related to social movement activities in the local community	[34]
		Interactive and interesting program	[34]
		Greater effort and commitment in knowing that the physiotherapist would be returning	[37]
		Ease of access	[40]
		No preparation needed	[40]
		Enjoyable	[40]
		Adaptation for people with PD and one’s individual abilities	[40]

**Table 6:** Overview of the barriers and facilitators of remote consultation.

**Category: remote consultation**			
**Barrier**	**Study**	**Facilitator**	**Study**
Related to clinicians (from the patient’s perspective):		Related to clinicians (from the patient’s perspective):	
- Concern that family physicians and general neurologists did not have the necessary expertise to manage their PD	[42]	- Access to high quality, dedicated PD experts	
- Shortage of expertise and neurologists in smaller centers	[45]	- Feeling that doctor listens to concerns	[42], [44], [45]
- Less confidence in physician	[44]	- More time with the doctor	
- Lack of access to multidisciplinary care and advanced therapies (e.g. DBS)	[45]	- More thorough assessment	[42], [44]
- Losing a personal connection with their healthcare provider	[41], [43], [44], [45]		
- Accessibility (not all persons with PD willing or able to use remote consultation)	[49]		[44]
- More teamwork of professionals necessary, each having clarifying roles and responsibilities	[49]		[44]
Technical issues (e.g. dropped signals, trouble hearing the doctor, inability to see the participants’ entire body, size of the tablet too small)	[42], [44], [46], [47], [48]	Convenience	[41], [42], [44], [46], [47], [48]
Acceptability among physicians	[42]	Easy communication	[42], [44]
Travel to the remote consultation site	[42]	Comfortable	[44], [48]
Mixed feelings about completing remote visits at home without medical and technical support	[42]	Related to traveling (e.g. no travel costs, no travel risks)	[47], [48], [41], [42], [43], [44], [45]
Long waiting times for initial consultations	[45]	No feeling of rush	[42]
Less comfortable	[44], [45]	Less strain on patient and caregiver	[44], [45]
Concern for others’ experiences	[47]	Ability to be assessed at home	[45]
Remote assessment feels incomplete	[48]	Treatment effectiveness	[46]
Barriers for healthcare professionals (their own point of view):	[49]	Opportunity to meet other persons with PD	[46]
- Additional administrative workload	[49]		
- Overuse of opportunity to report heath issues by persons with PD, increasing professionals’ workload	[49]		
- Lack of reliable means for determining rigidity, physical examination and the pull-test for postural instability	[50]		
Difficult communication	[41], [43], [44]	Time saving	[43], [44], [46]
Phone call more stressful than face to face contact	[43]	Appropriate frequency	[44], [46]
Lack of insight in body language	[43]	Efficiency	[41], [47]
No benefits versus usual care (e.g. when living close to physician or already satisfactorily engaged with a PD specialist)	[44]	Effectivity	[46]
Lack of continuity of care	[44]	Ability to display symptoms that are intermittent	[47]
		Positive feeling on interaction via teleconference	[47]
		Good connection	[48]
		Ease of scheduling	[48]
		Electronic pre-visit form	[49]
		Patient self-tracking of health	[49]
		Graphical overview of health data	[49]
		Clinical decision support functionality	[49]
		Provision of self-care recommendations	[49]
		Text-based messaging for asynchronous communication	[49]
		Useful as addition to face to face contact	[43]
		Better preparation of the appointment by the patient	[43]

### 3.1 Cueing – Results

Of the three articles that focused on cueing, one investigated the barriers and facilitators of cueing with a red, green and blue color depth (RGB-D) camera (Microsoft Kinect) [17] and two that used smart glasses [18], [19]. Low usability was reported as a barrier for both the RGB-D camera and the smart glasses [17], [18]. For the RGB-D camera, this was attributed to the device not being portable [17]. The smart glasses had a low usability for multiple reasons, amongst others because it was not comfortable due to its weight and size [18]. Furthermore, the visual cues as delivered by smart glasses were not optimal because they were found to be distracting as they were blocking the visual field [18], [19]. On the contrary, high usability was reported as a facilitator in all three articles as well [17], [18], [19]. Both the RGB-D camera and smart glasses were considered easy to use [17], [18], [19]. In addition, using the smart glasses was easy to learn [18] and the instructions that were presented on the screen were clear to read [19].

### 3.2 Cueing – Recommendations

Based on these results, some recommendations emerge to improve successful implementation of cueing as technology: the device should be portable [17], comfortable [18], and cues must not be distracting or blocking the visual field [18], [19]. Especially since cueing is used to improve walking capacity in people experiencing freezing of gait [14], and distractions may result in a more severe form of freezing of gait, and therefore have adverse effects [51].

### 3.3 Exergaming – Results

In the category of exergaming, thee different types of games were investigated. The first one was a rehabilitation game to train dynamic postural control using a RGB-D camera [20]. The second study used an interactive dance videogame to improve cognitive movement strategies, physical capacity, balance training and cueing [21]. The last game was a serious game using virtual reality technology for upper limb mobility [22]. Several features of the game were reported as the most important barriers in all studies [20], [21], [22]: the pace of the game was too fast [20], the music was not appreciated [21] or the game was monotonous [22]. Moreover, the costs of the games were found to be a barrier for actual use in daily life [20], [21]. On the other hand, some features of gaming were seen as important facilitators [20], [21], [22]. These included for example satisfying sound effects associated with actions [20], the ability to compete with other people (i.e. competition) [20] or the possibility to improve personal scores (goal setting) [22]. This all contributed to enjoyment of playing the game, which was reported as a facilitator on its own as well [20], [21].

### 3.4 Exergaming – Recommendations

Recommendations for a successful implementation of exergaming include the development of a multifaceted game with several levels of speed and difficulty [20]. In addition, the game should have the ability to personalize e.g. sounds, characters and environments [20], [21], it should have variable tasks to prevent it from being monotonous [22] and it should contain competition elements, since it is more appreciated when people can compete with others or improve personal scores [20], [22]. This will all contribute to a higher enjoyment, facilitating actual use in daily practice.

### 3.5 Remote monitoring using wearable sensors – Results

Ten articles focused on remote monitoring using wearable sensors, with several aims for using this technology. These included objective assessment and monitoring of motor symptoms of persons with PD [23], [24], [25], [26], [27], [31], [32], assisting in treatment decisions and evaluations [28], [30] and monitoring physical activity [29]. The visibility of the device was reported multiple times as a barrier for using wearable sensors [23], [24], [25], [26], [32]. That was even stronger among early-disease stage persons with PD [23]. In addition, persons with PD may experience discomfort or pain while wearing the device [24], [26], [28], or may have allergic reactions to the device [25], [29]. Furthermore, the device did not always feel comfortable [25], [26], [28], for example because the attachment was too tight [25], [26] or it was difficult to fasten the strap during OFF medication state [28]. On the other hand, persons with PD also reported not experiencing any harm whilst using the device [23], [24], [26], or finding it comfortable to wear [23], [28] as a facilitator. An additional facilitator was that the wearable sensor did not obstruct or interfere with activities of daily life [24], [26]. These all might have contributed to a high wearability, which was seen as a facilitator by itself [23], [25], [28]. Moreover, the use of wearable sensors was facilitated by the collected data that provided more insight into the health of persons with PD, both for the patients themselves [23] and for the healthcare providers [27], [30], [31], [32].

### 3.6 Remote monitoring using wearable sensors – Recommendations

The use of wearable sensors for remote monitoring can be facilitated for people with PD by using either a limited number of small sensors, or sensors integrated in commonly used accessories such as (smart)watches, because visibility causes issues with stigma [23], [24], [25], [26], [32]. Besides that, the sensors should be comfortable to wear [23], [24], [26], [28] and should not interfere with activities of daily life [24], [26]. The wearability should be good, as people with PD might experience difficulties with the attachment during OFF [25], [26], [28], and alternative materials must be considered due to the possibility of allergic reactions [25], [29]. For healthcare providers it is important to have access to data that is easy to interpret [23], [27], [30], [32]. These data should at least comprise medication intake, (night-time) activity levels and motor symptoms [27], [31]. Moreover, the use of wearable sensors will be of added value when healthcare providers have the ability to compare the data of different people [30]. When widely accepted and used properly, wearable sensors have the ability to support in clinical decision making [52] and in improving personalized care and PD management.

### 3.7 Telerehabilitation – Results

Eight studies on telerehabilitation were included, using a digital connection for home-based exercising [33], [35], [37], [39], an online dance therapy [36], [40], a home-based mindfulness yoga program [34], or a RGB-D camera to record in-home falls and movements [38]. Some negative experiences were reported as a barrier for using a video connection for telerehabilitation, including technical issues (e.g. internet instability) [35], [36], [37], [39], [40], but also the feeling of being watched when using a camera [38]. Besides that, participants experienced challenges during exercising at home due to PD symptoms [33], [37] or not having the same equipment and space as at the center [33], [37], [39], [40]. They also had some concerns about the correct execution of exercises and yoga postures [33], [34], [37]. Usability was seen as a facilitator for using video recording as the software was straightforward [35], [38] and preparation was not needed [40]. Also, the availability of a clear description of the program was very helpful [33], [37]. Some travel-related facilitators were mentioned as well, including not being bothered by travel issues and traveling in adverse weather [33], [34]. Participants of various studies liked to experience the beneficial effects, e.g. the improvements in self-confidence [36] and decrease in motor- and non-motor symptoms [33], [34], [40], but also practically in the flexibility to complete the exercises at a time that suited [33], [35] and the possibility of employing strategies into daily life [37]. Also, variety in dance genres and music facilitated the participation of the online dance therapy [36]. Moreover, using a RGB-D camera for recording in-home falls and movements was not seen as cumbersome, and it did not hinder or distract participants [38].

### 3.8 Telerehabilitation – Recommendations

In order to facilitate the use of telerehabilitation in daily life, the camera should be small and not too obviously present, as people do not like the feeling of being watched [38]. Additionally, the camera should not cause any hinder or distraction [38]. The use of the camera should also be easy [35], and access to technical support [36], [39] or a clear description [33], [37] should be available when using technology. Furthermore, when telerehabilitation is used for therapeutic purposes, the therapy must be challenging, but still appropriately tailored to the capability of persons with PD [36]. People like to experience the benefits of telehealth for an increased usability [33], [35], [36], [40], so insight in the collected data should be available by the users. Personalization and variation should be available to facilitate participation and prevent boredom of therapies [36], [37]. When telerehabilitation is used for exercises, it should preferably alternate with face-to-face sessions as well to make sure that the exercises are performed correctly [33], [34], [37]. Moreover, previous work has emphasized that privacy of participants should be taken into account as well during video recording [53], so the camera placement and moments of recording should be well considered for an increased usability in daily life.

### 3.9 Remote consultation – Results

This category of remote consultation included ten articles. These articles either focused on remote consultation compared to usual care [42], [44], [46], [47], remote consultation as an addition to usual care [43], [45], [48], [49], [50] or the use of remote consultation as result of the COVID-19 pandemic [41]. One barrier reported in multiple studies included the occurrence of technical problems [42], [44], [46], [47], [48], for example due to dropped signals or audio and/or visual difficulties [42], [44]. In addition, persons with PD had the feeling they were losing a personal connection with their caregiver [41], [43], [44], [45]. Furthermore, participants experienced more difficult communication via remote consultation [41], [43], [44], or found it less comfortable than a physical consultation [44], [45]. Most studies reported facilitators of remote consultation that were related to traveling [41], [42], [43], [44], [45], [47], [48], including no travel costs [45] and no travel risks [45], [48]. No traveling also contributed to time saving [43], [44], [46]. Virtual care also contributed to a more efficient consultation [41], [47]. Participants additionally experienced easy communication by making use of remote consultation [42], [44], [49]. Furthermore, they had the feeling that the healthcare professional had more time [44], did a more thorough assessment [44] and actually listens to the patient’s concerns [42], [44]. In addition, participants experienced less strain on the patient and/or caregiver by using remote consultations [45]. All of this also contributed to convenience, which was reported as a facilitator on its own [41], [42], [44], [46], [47], [48]. Besides that, an appropriate frequency of remote consultations was seen as facilitator [44], [46]. Moreover, multiple studies reported the ability to have access to high quality, dedicated PD experts by using remote consultation as a facilitator [42], [44], [45].

### 3.10 Remote consultation – Recommendations

For the use of remote consultation in daily practice, it is recommended to proactively tackle frequently occurring technical issues and to have a technical support team available to help users with technical problems [42], [44], [46], [47], [48]. In addition, even though the use of remote consultation might lead to a reduced travel time, less travel risks and lower travel costs [41], [42], [43], [44], [45], [46], [47], [48] and give more people with PD access to high quality, dedicated PD experts [42], [44], [45], it might also result in problems related to communication [41], [43], [44]. People might find it less comfortable to talk to remote professionals [43], [44], [45], although others find it more comfortable [44], [48]. The preference for remote consultation, and its frequency, is very personal and should therefore be discussed with each person separately.

From the perspective of the clinician, the use of remote consultation might result in a higher workload due to more administration or the overuse for patients to report health issues remotely [49]. Therefore, the additional administrative tasks should be reduced in the development and specific moments should be planned to respond on the reports to reduce this workload. Furthermore, there is currently a lack of reliable means for determining some of the PD-related signs, such as rigidity or postural instability [50]. Along with the fact that face-to-face contact is necessary for an adequate neurological exam and contributes to a more personal connection, it is therefore recommended to still execute face-to-face consultations as well, especially for the first contact with a new patient or healthcare provider [43], [44], [45]. A good solution might be to alternate of face-to-face consultations with remote consultations. Although the use of remote consultation has grown during the COVID-19 pandemic, it has become clear that it will never replace face-to-face consultation in the field of neurology [54]. However, when used correctly, it has the ability to complement in-person visits and – importantly – to provide a reasonable alternative for the many persons with PD in the world who have no access to in-person visits at all.

## 4 Discussion

Here, we reviewed the experiences of persons with PD and their healthcare providers with technologies and innovations. By combining the outcomes of multiple qualitative studies, we were able to provide a clear overview of important factors that should be considered during further development and implementation of technologies to better manage PD. The importance of technology for personalized care is already well established [55]. Moreover, the current COVID-19 pandemic has further emphasized the importance of implementing these technologies, as they can be used to ensure the continuity of care while also reducing personal contacts and mitigating the risk of infection [56]. We hope that our present results may help to accelerate the implementation of technology in PD care. As next steps, we recommend to further test technologies in larger groups of people with PD, aiming to establish their actual added value in daily clinical practice. On the other hand, we also recommend to always include the perspectives of people with PD (and if applicable their caregivers or healthcare providers) right from the start while developing and testing technological innovations.

The most important barriers and facilitators as found across categories show similarities with other studies in the field of neurology [57], [58]. For example, people with amyotrophic lateral sclerosis valued the user-friendliness of devices, the feeling of security whilst using it and the improved feeling of confidence as a result of it [57]. This all contributed to a positive attitude towards telehealth, which was an important facilitator for implementation. Moreover, usability contributed to high satisfaction of using technology in people with multiple sclerosis, stroke and PD [58]. Furthermore, in people with neurological disabilities, exergaming is as good as traditional exercise, but more enjoyable [59]. Therefore, games will help them to adhere to the physical activity guidelines of the World Health Organization [60]. When this effect can be reached in people with PD, their physical- and functional capacity are likely to improve [61], contributing to a higher level of independence and therefore a higher quality of life [13]. In our review, the enjoyment and relevance of the intervention for everyday life were important facilitators for people with PD as well. On the contrary, the most important barrier for using telehealth in the field of neurology were technical issues [57], [58] and costs of the intervention [57]. When working with people with PD, special attention must be paid to the PD-specific (motor) symptoms that might hamper the usability of technologies. These are not, or to a lesser extent, described in other fields of neurology but do contribute to a lower usability of technology and are thus a barrier for its implementation.

Some critical notes must be mentioned about our review. Most importantly, articles evaluating user experiences with technology qualitatively are scarce. The effects of using technologies is widely studied, however, to make it work in daily practice, it is important that the technology meets the wishes and needs of the users. Consequently, we decided to include all relevant articles, without any selection based on quality. Also, the number of participants in the included articles was limited potentially leading to selection bias. Large-scale studies in a representative group of PD patients remain needed to further develop and test the technologies together with end-users to facilitate the implementation in daily practice. Furthermore, most included studies only focused on the experiences by PD patients. This is, of course, important as they are primary end-users. However, when the technology is used to give insights into disease development for e.g. healthcare professionals as well, their experiences should be also be accommodated to further develop technologies. In addition, the experiences of caregivers are important as well, but have not been systematically studied yet.

The barriers and facilitators identified in this study can be used to further shape future technologies for PD management. Concretely, this means that users should first become familiar with the technology before using it in daily practice, the costs should be reduced or reimbursed, technical issues should be tackled, a technical support team should be available and the known PD-related (motor) symptoms should not hamper the usability of the intervention. Furthermore, the intervention should not involve any risks and people using the intervention should be able to experience the beneficial effects by themselves to enhance the usability. For each category there are specific barriers and facilitators that should additionally be taken into account. Moreover, depending on the aim of the technology, people with PD, their caregivers or healthcare professionals should be involved in developing the technology. This way, the drawbacks can be addressed directly and facilitators can be emphasized. As a result, the intervention is more likely to be implemented and used in daily life. Technology can be very important to improve the quality of life in people with PD, but they should match the wishes and needs of the users. When they do, the implementation of these fine-tuned technologies can be facilitated and ultimately be used to improve the (quality of) care in people with PD.

## Declaration of Competing Interest

The authors declare that they have no known competing financial interests or personal relationships that could have appeared to influence the work reported in this paper.
