# Supplementary material for: Successful implementation of technology in the management of Parkinson's disease: Barriers and facilitators
Source: Clin Park Relat Disord. 2023 Feb 16;8:100188. doi: 10.1016/j.prdoa.2023.100188 (PMC9972397; doi:10.1016/j.prdoa.2023.100188)
Supplement: Supplementary data 1 [file mmc1.docx]

| **Online database** | **Search terms** | **Studies** |
| --- | --- | --- |
| Pubmed | (requirement*[Title/Abstract] OR barrier*[Title/Abstract] OR limit*[Title/Abstract] OR enabl*[Title/Abstract] OR facilitat*[Title/Abstract] OR challeng*[Title/Abstract] OR opportunit*[Title/Abstract] OR success*[Title/Abstract] OR implement*[Title/Abstract] OR adaption[Title/Abstract] OR usability[Title/Abstract] OR feasibility[Title/Abstract] OR ease-of-use[Title/Abstract] OR feasibility studies[MeSH Terms])  AND  (ICT[Title/Abstract] OR (Information[Title/Abstract] AND Communication Technolog*[Title/Abstract]) OR technolog*[Title/Abstract] OR e-health[Title/Abstract] OR telehealth[Title/Abstract] OR remote monitor*[Title/Abstract] OR sensor*[Title/Abstract] OR telecare[Title/Abstract] OR telemedicine[Title/Abstract] OR telemonitor*[Title/Abstract] OR wearable*[Title/Abstract] OR assessment, technology[MeSH Terms] OR telemedicine[MeSH Terms])  AND  (Parkinson Disease[MeSH Terms] OR Parkinson*[Title/Abstract]) | 2589 |
| Embase | (requirement*.ti,ab,kw. or barrier*.ti,ab,kw. or limit*.ti,ab,kw. or enabl*.ti,ab,kw. or facilitat*.ti,ab,kw. or exp facilitation/ or challeng*.ti,ab,kw. or opportunit*.ti,ab,kw. or success*.ti,ab,kw. or implement*.ti,ab,kw. or adaption.ti,ab,kw. or usability.ti,ab,kw. or feasibility.ti,ab,kw. or ease-of-use.ti,ab,kw. or exp feasibility study/)  AND  (ICT.ti,ab,kw. or (Information and Communication Technolog*).ti,ab,kw. or technolog*.ti,ab,kw. or e-health.ti,ab,kw. or telehealth.ti,ab,kw. or remote monitor*.ti,ab,kw. or sensor*.ti,ab,kw. or telecare.ti,ab,kw. or telemedicine.ti,ab,kw. or telemonitor*.ti,ab,kw. or wearable*.ti,ab,kw. or exp information technology/ or exp technology/ or exp telehealth/ or exp telemonitoring/ or exp sensor/)  AND  (Parkinson*.ti,ab,kw. or exp Parkinson disease/) | 5159 |
