# Supplementary material for: Successful implementation of technology in the management of Parkinson's disease: Barriers and facilitators
Source: Clin Park Relat Disord. 2023 Feb 16;8:100188. doi: 10.1016/j.prdoa.2023.100188 (PMC9972397; doi:10.1016/j.prdoa.2023.100188)
Supplement: Supplementary data 2 [file mmc2.docx]

| **Category: cueing** | |
| --- | --- |
| **Study** | **Other outcomes** |
| Amini et al.  (2019) | Suggestion for further improvement:   - Telephone call or Skype video call as remote communication method between healthcare provider and patient during a critical fall incident. |
| Janssen et al.  (2017) | Suggestions for further improvement:   - Pilot with softer colors than white; - Broaden augmented cues; - Include footsteps on the augmented staircase cueing type.   User experience:   - Conventional metronome was preferred most, followed by transverse conventional bars, augmented transverse bars, no cues and augmented staircase; - Participants reported better walking with cues; - All cues except augmented staircase made walking easier; - All cues except augmented transverse bars were considered useful; - Conventional metronome was considered most well-suited cue to provide more control over daily life activities, and met participants’ needs and expectations most; - Augmented transverse bars and transverse conventional bars were less distracting than augmented staircase and conventional metronome. |
| Zhao et al.  (2016) | User experience:   - Self-reported improvement on walking; - Metronome was the most effective and most preferred; - Optic flow was the least preferred; - Synchronizing to the rhythm of the cues and walking while focusing on the cues was easier whilst using the metronome than whilst using the optic flow.     Visual and auditory cues compared to no-cues:   - No statistically significant difference was found in the number of Freezing of Gait (FOG) episodes and FOG duration among cueing conditions. - Fewer participants experienced FOG whilst using any cue; - Statistically significant less FOG episodes occurred per trial while using the metronome compared to no cueing; - A statistically significant decrease in stride length variability for all cues was found; - Metronome was associated with a statistically significant increase in speed only during doorway course; - Optic flow and LED were associated with a statistically significant decrease of speed during the wide and narrow turn courses; - All cues showed statistically significant decrease in cadence for the narrow and full turn courses; - LED was associated with a statistically significant decrease in cadence during the wide turn course; - No statistically significant effect in cadence was found for any cue during the doorway course; - Cadence tended to cluster around the cueing frequency for the three cues type used; - Cadence was less variable in its distribution for the metronome and optic flow. |
|  |  |
| **Category: exergaming** | |
| **Study** | **Other outcomes** |
| Galna et al.  (2014) | Part 1:   - Participants reported difficulty in repeatedly stepping on the Nintendo Wii Fit™ balance board as part of a dancing game; - Using the handheld Nintendo Wii™ controller was frustrating when playing a golf game; - Twelve levels of increasing complexity were available to prevent the game being too complex at first; - Postural control was trained under dual-task conditions since persons with PD have difficulties performing motor tasks when dual-tasking; - Game speed could manually be adjusted by either player or clinician to ensure an appropriate game speed.   Part 2:   - Participants already played games at home, with most of them playing paper based puzzles such as Sudoku or crosswords; - Some participants played Nintendo Wii™ at home; - Most patients were self-motivated to exercise; - All patients exercised at least once a week; - Walking was the most common type of exercise; - No adverse events as result of the game, except one participant feeling dizzy as result of hypotension; - Levels were too demanding for 33.3% of participants. |
| Natbony et al.  (2013) | - All patients have performed physical activity in their lifetime, but currently none of the included patients did; - 50% of the participants had prior experience with videogames; - Borg scale (0-10) for pain was 0 for all participants; - Borg scale (0-10) for distress and fear was ≤3; - Borg scale (0-10) for discomfort was 0 for all but one participant (rating it a 3); - Perceived exertion was 1.1 (0.8); - No reports of loss of balance or need to prevent a fall. |
| Sanchez-Herrera-Baeza et al.  (2020) | Part 1 (Quantitative data collection with the OR2-LMC intervention):   - High degree of satisfaction was found according to the client satisfaction questionnaire (CSQ-8): mean score of 3.66 (±0.18) points out of the maximum of 4; - Highest scores were given to the question “Are you satisfied with the help you have received?” and “In general, are you satisfied with the services you have received?” by all participants; - No disagreement or dissatisfaction was reported in the remaining questions by any of the patients; - Attendance rate was 100% for therapy sessions; - No adverse side effects were observed in the intervention.   Part 2 (Semi-structured interviews):   - All patients self-reported that the OR2-LMC treatment was not better at increasing their upper limb functionality than the conventional treatment (physiotherapy, occupational therapy, etc.); - The OR2-LMC treatment did not replace conventional treatment, but rather complemented it; - Health professionals reinforced the importance of movement-oriented treatments and the avoidance of immobility; - The new treatment was for some patients more mentally than physically challenging; - No participants spontaneously reported any improvements following OR2-LMC treatment; - Some patients perceived improvements in daily activities, e.g. eating, handling utensils, buying food, checking tickets at home, driving, looking at traffic lights on the street and reading. - Some patients perceived improvements in coordination, joint movement, concentration, mental speed during activities, and the ability to overcome obstacles on the street. - There was a process of adaptation of the new treatment by participants (patients felt awkward when trying to adapt to the “virtual world”, although they did gradually become more comfortable and improved their performance); - Treatment seemed inapplicable to the home setting (system requires a lot of physical space, is complex to assemble, demands previous knowledge and skills for use, and requires a lot of time and money); - Treatment should be administered by a qualified professional (in order to prepare and operate the equipment and resolve any unforeseen events that may occur); - Professional support was needed to monitor and track the results of the treatment, or to correct actions of participants and help guiding them through the correct performance; - Some patients preferred to be monitored by a health professional (due to feeling of safety, as well as the inability to postpone treatment or falsify their results), although they felt their families could help them if necessary. However, family members should not be responsible for applying the treatment.   Suggestions for improving the game:   - Implement competition among the users; - Conduct preparatory sessions to increase familiarity with VR before treatment; - Expand the catalogue of games and activities available to increase motivation and help with the treatment of additional symptoms of PD, such as tremor and lack of balance; - Clearly explain the treatment, its application, and realistic expectations of results during recruitment to avoid people not fully understanding what the treatment involved or having unrealistic expectations of potential improvements; - Include various levels of difficulty in the video games to stimulate continuous efforts; - Require treatment administrators to experience the treatment before applying it in order to first gain experience with the technology. |
|  |  |
| **Category: remote monitoring using wearable sensors** | |
| **Study** | **Other outcomes** |
| AlMahadin et al.  (2020) | - Current assessment of PD-related symptoms were subjective, dependent on clinical expertise and thus inconsistent; - There was much scope for Type I (false positive) and Type 2 (false negative) errors in current diagnosis; - Current assessment and monitoring processes were dependent on patients’ memories and diaries which are not reliable; - Many PD patients were unaware of their symptoms and often cannot distinguish between PD signs and other abnormal activities; - PD patients were often not familiar with modern technologies, which made it difficult to ask them to make a video of tremors they are experiencing at home; - Healthcare professionals had high interest in continuous monitoring and its importance to diagnosis and treatment decisions; - Remote monitoring had a pivotal role in PD treatment as well as healthcare cost reduction, mainly for large geographical areas; - Remote monitoring was easier and more efficient use of time for healthcare professionals to assess patients remotely rather than traveling to their homes; - Remote monitoring and assessment could lead to early precise diagnosis and accurate frequent assessment which can make life easier for the patient and might lead to better treatment. |
| Cancela et al.  (2013) | - The technical performance of the system showed an accuracy of 93.73% for the classification of levodopa induced dyskinesias severity, a 86% of bradykinesia severity and 87% for tremor; - A special module was developed for the assessment of the gait parameters (i.e. step frequency, velocity, arm swing frequency and entropy of the gait signal); |
| Cancela et al.  (2014)  Wearability | Physiological:   - The wearable device was an extra load that gets attached to the human body, which could have an effect on both the energy consumed by the body and the result in movement and posture changes; - Most patients did not feel any exertion caused by the device; - One patient (a 71 year old female) reported a moderate feeling of physical stress by using the device.     Biomechanical:   - The device could cause musculoskeletal loading, which might result in perceptions of muscle fatigue; - The wearable device did not have any impact on the patients’ posture.   Comfort:   - Both muscle fatigue and musculoskeletal loading might lead to discomfort due to stresses placed on specific physiological systems or affect the general sense of well-being; - The social component was a concern since some patient expressed their worries about what other people could think about them; - The anxiety caused to patients by the device was generated by a lack of feedback about the correct placement and functioning of the device. |
| Cancela et al.  (2014)  Feasibility | - The average data loss was minimal; in all cases more than 95% of the data was successfully transmitted. |
| Elm et al.  (2019) | - The total number of hours of accelerometer data streamed from participants was 83,432 (91% of expected); - Reporting of symptom severity was lower than expected, 44% overall, with an average of 8 daily reports of symptom severity; - The average compliance with device usage was 66% (SD = 34%); - Compliance level was similar across demographics and PD characteristics (MDS-UPDRS, MoCA, PDQ-39), although there was a marginal difference by age and enrolling site, which did not reach statistical significance; - The correlations between MDS-UPDRS part 2 items and electronic patient-reported outcomes (ePROs) (averaged across the week prior to the in-office visit) were moderate.   Suggestions for further improvement:   - The creation of separate displays for ePROs and sensor-derived data display; - The expansion of the Y-axis in certain displays for easier data comprehension; - The addition of markers for medication intake across ePROs and sensor-derived data displays; - Updating the descriptive text to make the dashboard easier to understand; - The addition of “info” buttons describing each display. |
| Fisher et al.  (2016) | - 608/612 (99.3%) responses to the questionnaire items from both phases were completed, with only four invalid responses (three blank, one dual-selection); - One participant (who withdrew due to sensor discomfort) reported a preference for keeping a symptom diary as opposed to wearing the sensors; - 32/34 (94.1%) participants agreed that they were willing to wear the sensors at home; - 29/34 (85.3%) participants agreed that they were willing to wear the sensors in public; - A statistically significant change (toward less agreement) in the responses was found in Items 1 (the sensor looks like it is well made), 2 (the sensor is comfortable to wear), and 5 (I would be happy to wear the sensor around the house); - The majority of participants showed no change in their responses over time; - For participants whose responses declined in agreement, it is evident that the majority did so only by one category, with more pronounced swings in opinion (change by two or more categories) being rare; - After using the device for one week, a change in opinion of two or more categories was only expressed by 2/34 (5.9%) of participants to Item 1, 3/34 (8.8%%) to Item 2, and 2/34 (5.9%) to Item 5; - There was no significant difference between the study phases for the responses to the remaining five items considered for both phases of the study; - For Items 1, 2, and 5, further analysis was undertaken following contraction of the 5-point Likert scale into a 3-point scale, resulting in no statistically significant change in participants’ responses between study phases for Items 1 and 5. However, it did result in a statistically significant decrease (toward less agreement) in the responses to Item 2; - The mean duration of ‘‘non–wear time’’ was 32.6 min/day. |
| Hermanns et al.  (2019) | - All comparisons among the four pre-test and post-test Functional Assessment of Cancer Therapy-General (FACT-G) subscales and the two FACT-G and Physical Activity Assessment Inventory (PAAI) total scores using Wilcoxon signed-rank tests resulted in nonsignificant (p > .05) findings; - Percentage of change found suggests that the physical activity tracker and electronic tablet intervention did not have a significant effect on participants’ Quality of Life or self-efficacy for physical activity; - The majority of participants’ scores on quality of life decreased following the intervention, with the FWB subscale decreasing the most at 5.83%; - The FACT-G total score decreased 3.18% from the preintervention surveys to the postintervention surveys; - The PAAI total score also decreased considerably at 42.86%, suggesting lower self-efficacy for physical activity following the intervention; - An evaluation of the data analytics from physical activity tracker demonstrated that only one participant’s activity level increased from preintervention to postintervention; - Participants required some time to become acclimated to the physical activity tracker and the electronic tablet; - Examination of the data retrieved from the physical activity tracker and electronic tablet demonstrated that four out of five participants were willing and able to wear the activity tracker and all five were willing to participate in the online support group; - In this study, participants shared responses that were not only inspiring but also reflected courage and hope among fellow group members; - Two of the group members encouraged others to maintain their mobility. - Support was most prominent with regard to use of the physical activity tracker and physical activity; - Many of the participants posted their daily exercise; - Support was evident when one participant posted about a technical issue encountered with the physical activity tracker device; - All participants who wore the tracker provided a “yes” response when asked whether the physical activity tracker made them more aware of their physical activity level. |
| Memedi et al.  (2011) | - A majority of the clinics were quite satisfied with the usability although a sizeable minority were not; - Two out of seven clinics answered that they had much previous experience with computers, whereas four had some experience and one had little experience. |
| Timotijevic et al.  (2020) | Part 1 (Hierarchical Task Analysis):   - The outcomes of this study suggested a move away from the traditional silos of ‘motor’ and ‘non-motor’ symptom evaluations and suggest that presenting data on symptoms according to goal-based domains would be the most beneficial approach; - The data suggested that the most useful symptoms to evaluate should be driven by the importance the patient themselves attribute in terms of their overall Quality of Life; - The content analysis of attitudes towards the technology and ethical issues indicated that the participants were not aware of any ethical issues associated with the mHealth-based Clinical Decision Support Systems.   Part 2 (Conceptual modelling):   - Across the different models, clinicians were more likely to change the care plan and increase the level of medication when presented with worsening symptoms, with the exception of a reduction in Levodopa with worsening dyskinesia and a reduction in dopamine agonist with worsening impulsivity; - Across the different judgements, the factors that dominate the decision were motor symptoms: bradykinesia; rigidity; motor fluctuations; and tremor; - Non-motor symptoms such as depression, impulsivity and cognitive function explained some variance in the judgements, albeit less than bradykinesia, rigidity, motor fluctuations and tremor, but sleep and constipation had very little effect on the judgements made; - Some factors were weighted more similarly by all clinicians, e.g. tremor, whereas others were weighted more differently, e.g. bradykinesia; - The study showed that different factor combinations (e.g. symptom, age, employment status) are important when making judgments about different questions (e.g. changing the care plan, change to Levodopa, and change to dopamine agonist).   Part 3 (Vignette study):   - The clinicians were equally likely to change the care plan based on information from the patients’ self-report and the devices about the decline of their condition; - The clinicians were more likely to revise the care plan when they received congruent information from both sources and less likely to revise the care plan when they received conflicting information from both sources; - Clinicians were equally confident in changing the care plan when receiving information from only one information source, most confident when the information from both sources was congruent and least confident when it was incongruent. |
| Virbel-Fleischman et al.  (2022) | Persons with PD:   - Each Body-worn sensor (BWS) was appraised by seven patients using the SUS and the AttrakDiff questionnaires (one missing data). No correlation was found between age or disease duration and the SUS and AttrakDiff scores; - According to the SUS results, the global usability score for BWS is 87.5 [72.5; 100] (median [range]). The three BWS were assessed as "excellent" to "best imaginable" usability (all scores were above 72.5 on the 100 point scale). SUS scores of BWS a, b, and c were comparable: 90 [77.5; 97.5], 87.5 [72.5; 92.5] and 95 [82.5; 100], respectively; - The four categories of the AttrakDiff questionnaire were assessed positively. Global scores for the three BWS regarding attractiveness, PQ, identification of the user to the BWS and stimulation by the BWS to the user were respectively (median [range]): 1 [-3; 3], 2 [-3; 3], 0 [-3; 3] and 2 [-3; 3]. The rating of the three BWS was positive for 84% of all AttrakDiff items, all categories combined. The three BWS yielded independently very similar results for each AttrakDiff dimension; - According to the two questionnaires and the phenomenological approach analyses, the three groups of patients were equivalent, and the patients’ descriptions allowed us to make general conclusions on BWS monitoring; - Before this study, 20 out of the 22 patients had not heard of BWS. Finding a new avenue to help patients with PD was the driving force of volunteering, as a majority of patients expressed. The progress inspired by the new term "BWS monitoring" was perceived as positive and the objectives attractive ("Carrying out this type of investigation is very serious."); - Patients developed creative thinking about sensors, and many questions emerged from their reasoning linked either to their interests in the device or to device functioning. During the first interview, the expressions used before having seen the BWS reflected positive feelings, interests, direct expectations in the patient’s life, and hopes in potential PD management advances. Negative feelings, such as anxiety about handling the device or doubts regarding technological capabilities, were little expressed; - All 22 patients expressed interest in BWS monitoring. The usefulness of the tool triggered expectations and hopes for disease follow-up; - According to 19 patients, clinicians would benefit from patients wearing the devices, as they would be able to adjust the treatment according to the results of BWS monitoring; - Eighteen patients felt the need to know more about their symptoms and 19 expressed the interest to get the results, either to justify the monitoring or their feelings to the physician and then improve the discussion (for 17 patients), or to better understand their disease (for 16 patients); - For 18 patients, the general exchange during consultations was incomplete because they could not have the time to discuss everything they wanted. In parallel, respectively eleven and nine patients were not satisfied with their treatment and PD follow-up at the time of the study. Finally, twelve patients also thought about research and the advantages of BWS monitoring for PD knowledge.   Healthcare professonals (HP):   - A significant interest in BWS monitoring was highlighted by all HP. |
|  |  |
| **Category: telerehabilitation** | |
| **Study** | **Other outcomes** |
| Flynn et al.  (2022) | - All participants reported that exercise was important; - Support from others including the physiotherapist, physiotherapy students and family/caregivers was important to help them to exercise; - None of the participants identified the exercise self-management program as providing support, with many having difficulty remembering this aspect of the intervention; - When exercising at the center in a group, participants reported developing a sense of community which reduced the feeling of isolation some people experienced; - Participants saw the same people each session providing an opportunity for social interaction before, during and after the class; - When exercising at the center the participants compared themselves to others in the group. This comparison provided them with a broader context of Parkinson’s disease and how others managed their condition. It also gave them a different perspective on the impact of Parkinson’s disease on their lives compared to others. However, for those who were younger or more recently diagnosed, exercising in a group with people who had more severe Parkinson’s disease was an uncomfortable and confronting experience. |
| Kwok et al.  (2022) | - The compliance rates for the daily 15-min mindful walking self-practice during the intervention period were 100%; - The mean (SD) frequencies of mindful walking were 1.8 (0.2) times per day; - Participants showed a significant improvement in functional balance (BBS: mean change=5.13; p < 0.001) from baseline to 1-week follow-up; - Participants showed a significant improvement in overall motor symptoms (MDS-UPDRS-III: mean change=12.63; P=0.002) from baseline to 1-week follow-up; - For subjective measures, perceived balance confidence during the “on” (ABC-ON: mean change=3.63; P=0.28) and “off” states (ABC-OFF: mean change=-13.22; P=0.14) and perceived Freezing of Gait (FOGQ: mean change=1.38; P=0.23) were insignificant; - For psychological distress, participants showed a significant reduction in anxiety (HADS-anxiety: mean change=9.00; P=0.002) and depressive symptoms (HADS-depression: mean change=3.63; P=0.04). - For mindfulness, an improving trend was noted regarding the domain of “nonjudging to inner experience” (FFMQ-non-judging: mean change=-2.25; P=0.05). Health-Related Quality of Life showed insignificant improvement (PDQ-8: mean change=0.78; P=0.82); - The adherence rate of online intervention was 98.4%, in which one participant missed a class due to a schedule clash with medical appointment; - The mean (SD) of the Borg CR10 was 5.68 (1.03) with a range of 4 to 8, indicating the safety and moderate intensity of the exercise; - All online sessions demonstrated a satisfactory treatment fidelity; - The self-practice of mindful walking had a mean (SD) time of 23.13 (12.64) minutes per day and ranged from 10 to 60 min per day; - All participants attended the pre- and post-intervention follow-ups. |
| Lai et al.  (2020) | - Participants in the Telecoach-assisted exercise (TAE) group achieved high rates of attendance (99.2%) and on average performed more exercise sessions than the total 24 sessions prescribed (a few participants exceeded the prescription); - Compared with the TAE group, self-regulated exercise (SRE) participants demonstrated:   - 36.1% lower attendance;   - 36% fewer total sessions;   - 48% less total time exercising;   - 74.5% less time performing moderate aerobic exercise. - Participants in the TAE group achieved:   - a mean change in 6MWT of 35.3 ± 30 meter;   - a mean change of -0.31 ± 0.77 m/s in 10 meter walk comfortable speed;   - a mean change of -0.16 ± 0.63 m/s in 10 meter walk fastest speed. - In the SRE group:   - the mean change in 6MWT was 1.35 ± 50 meter;   - the mean change in 10 meter walk comfortable speed was 0.11 ± 0.67 m/s;   - the mean change in 10 meter walk fastest speed was 0.002 ± 0.41 m/s.   Suggestions for further improvement:   - Six TAE participants identified that Internet instability caused frequent disconnects in communication between telecoach and participant and noted that the technology required a learning curve. |
| Morris et al.  (2021) | - Attendance was high (dancers attending 100% sessions); - Compliance to protocol was excellent (all except one post-class survey completed by dancers and all surveys completed by dance instructors); - No safety issues or adverse events; - Many participants had participated in face-to-face dance classes and were keen to pursue online delivery one-to-one with a dance instructor during the COVID-19 pandemic. |
| Roswell et al.  (2022) | Results of the initial interviews (prior to the home-based intervention):   - Nearly, all had experienced physiotherapy in the past and just under half had experienced other forms of rehabilitation and complimentary therapies (including reflexology, acupuncture, speech therapy, and massage); - Around half of the participants had taken part in structured exercise, such as classes at their local Parkinson’s group; - In the initial interviews, the majority of participants correctly anticipating that the PDSAFE intervention would include an element of exercise, which could be done at home and was designed to help them with their mobility. This could be attributed to information included in the Participant Information Sheets, the consent process for participation in the main trial plus wide experience of rehabilitation both for Parkinson’s and other conditions. A smaller number of participants talked in greater detail about what this might involve; including tailored exercises, stretching and exercises for balance, co-ordination, posture, freezing, stiffness, and muscle tone. Occasionally participants talked about being “assessed” or “monitored”; - The majority of participants were pleased that the PDSAFE program was home-based; reasons for this included convenience, problems travelling due to mobility, not having to visit hospital sites or clinics or park a car. - In terms of anticipating benefits from the program, most participants placed particular emphasis on projected improvements in physical functioning and mobility, including improved balance, increased strength, enhanced co-ordination, stability, and posture. Participants described expecting to be provided with strategies to help them manage their Parkinson’s; - Some participants spoke of very specific things they wanted help with or to achieve; for example, everyday activities such as putting on their socks, manipulation of buttons, getting up from a chair, crossing the road or getting in a lift; - Participants hoped the program would help boost their confidence, and support their social contacts, for example with family; - In general, participants at the initial interview were looking forward to taking part in the program and valued the opportunity to learn from a trained Parkinson’s physiotherapy specialist about suitable and tailored exercises for them; - A small minority of the sample expressed feelings of anxiety about the early therapy sessions, in part related to perceived anxieties about what the program might involve and the intensity of the sessions; - Some participants said they had not previously experienced success with physiotherapy and so wondered how beneficial it might be this time round.; - There were also a few participants who did not know what to expect from the PDSAFE program and had little idea about what it might involve for them.   Results of the follow-up interviews (after completing the home-based intervention):   - Most people found the level of the program acceptable and generally recognized the progressive nature of the program (i.e., increased in intensity and number of repetitions); - A small minority of participants found the exercises too easy (especially younger participants) or too challenging (particularly older participants); or occasionally felt they were aimed only at older people; - Most participants acknowledged that there was an expectation for them to carry out the exercises and program almost daily (or as agreed with their therapist); this was challenging for a number of participants; - Amongst the minority who used the weighted vests, there were mixed views; some users “loved it”, found it useful and felt it made the exercises more challenging for them; this was especially true for younger people and those whose Parkinson’s was not severe. Others disliked the weighted vests, describing them as not being very practical, not coping with them, or not being useful; - A minority of participants felt that the DVD prompted or helped them to engage in the exercises; - More commonly participants described at least one problem with the DVD, metronome or filming; the metronome particularly was found to be hard to use, with participants experiencing difficulties in keeping in time; - Older people found the metronome particularly difficult to use; - On a few occasions participants accessed the metronome on an app on a portable device (such as a mobile phone), which was more acceptable and easier to use; - Although participants made reference to being filmed (excerpts from which were then used within the DVD to educate and prompt), they did not tend to use these DVDs at home; - A sizeable minority of participants expressed dissatisfaction that the equipment (other than their personal log sheet) was reclaimed at the end of the program; - Most participants felt they had experienced benefits as a result of engaging in the PDSAFE program including enhanced mobility through use of strategies such as slowing down and use of swaying to initiate movement, functional gains, psychological and social benefits such as improved confidence, prevention of falls and increased awareness of limitations and triggers for falls; - People valued the personalized nature of the PDSAFE intervention; - A minority of participants commented that the benefits they had experienced were limited, with some reporting no subjective change to their balance, mobility, or falls; - Several participants questioned whether the intervention could make any difference to them in the face of a deteriorating condition, even if they felt the program was in itself well designed. |
| Torriani-Pasin et al.  (2022) | - The average participation was 20.7 ± 14.9 sessions (47.1 ± 33.8%, 95% CI 14.0–27.4 sessions); - One participant (5.3%) skipped all sessions; - Every participant finished each exercise session; - Five (26.3%) participants attended at least 80% of all sessions; - Six (31.5%) participants attended <20% sessions. - Active participants did 95.0 ± 21.6 min/week of physical activity with this program; - Total sessions were 394, accounting for 23,640 min of physical activity; - A Kruskal‐Wallis one‐way ANOVA showed no differences in participation for severity (*H*(1,18) = 0.01, p < 0.74); - For FOG, one‐way ANOVA showed no differences in participation for participants with or without FOG (*F*(1,18) = 3.6, p = 0.73); - Participants with a family member or a caregiver assisting them had higher attendance (35.1 ± 7.2 sessions) than participants without personal assistance (14.0 ± 12.5 sessions) (*F*(1,18) = 14.3, p = 0.001). - Most of participants reported at least one type of complaint that could be associated with safety (15 participants, 78.94%); - Participants reported pain during the sessions (13 participants, 55 sessions, 14.0%), and pain presented on the day after the session (10 participants, 62 sessions, 15.7%); while most of who felt pain during the session reported pain on the next day (9 participants, 69.23%). Only one participant reported pain on the next day without feeling pain during the session and four participants reported pain during the session without feeling pain after the session; - One participant reported one episode of fall (1 participant, 1 session, 0.3%). Regarding other safety issues, we had the following results: dizziness (6 participants, 19 sessions, 48%), motion sickness (1 participant, 1 session, 0.3%), and felt fear of exercising (7 participants, 49 sessions 12.4%) during the exercise sessions. The participant who reported fall episode did not complaint about dizziness, motion sickness or fear of exercising; - Only four participants did not present complaints, but their adherence rate was between 2.08% (1 session) and 14.58% (7 sessions). |
| Stack et al.  (2016) | - Researchers spent approximately seven hours with each participant, during which they were all largely sedentary (staying downstairs throughout the day, mostly in one favorite chair); - Participants predominantly used the furniture or walls for support (rather than mobility aids or purposely fitted rails) as they showed us around their homes and gardens and demonstrated the following activities: 1) Walking between rooms; 2) Preparing drinks or cooking; 3) Sorting, washing, and hanging out clothes; 4) Ascending and descending stairs; 5) Negotiating steps between rooms; 6) Crossing open spaces in large rooms; - No participant fell when researchers were present but they observed near-misses and remained vigilant throughout; - Participants frequently recounted falls with a sense of humor and told us we were being overcautious; - Two participants avoided using any support despite severe instability, even when demonstrating an activity associated with previous fall-events (which appeared the only physically demanding aspect of the study, though every participant was willing to do it); - The researchers greatest concern about the wearables and the RGB-D camera was the uncertainty about whether the devices were recording diverted their attention from the participants. - In the total period of 246 minutes of participant activity reviewed, 227 occasions when a participant appeared at imminent risk of falling were count; - All participants used support (mostly furniture) to preserve their balance; particularly when turning or rising, participants paused and either repositioned their hands or feet or aborted the action; - Participants flexed or rotated their trunks markedly to use every available support on the stairs; - Participants appeared particularly unsteady during turns and on steps, if they started to walk immediately on rising, and if they did not use support when standing or sitting down; - Balance was often lost backwards, but, in walking, participants tended to stumble forwards or sideways when their feet did not clear the floor or crossed, or they tripped or froze; - Unsteady transfers were characterized by swaying backwards (so that the toes lifted on standing) or by actually falling backwards into the chair (either on rising, or so violently during sitting that both feet lifted off the floor: twice a participant nearly missed the chair); - Participants unsteady walking or standing mostly took recovery steps, grabbed something, or sat down quickly to restore their balance; - When participants were unsteady transferring, the chair broke the potential fall, though on five occasions (three during transfers) another person assisted/caught the participant; - The participant’s histories, behavior, and thoughts, alongside the researchers’ observations, suggested that monitoring five activities could identify balance protection, loss, and recovery:   - Chair transfers;   - Walking (through open spaces and around furniture);   - Turning (in standing and walking;   - Stepping onto, off, or over obstacles/steps;   - Performing tasks in standing (e.g., conversing, cooking). |
| Walton et al.  (2022) | - In terms of technical and safety issues, 96% gave an average to positive score when considering how the digital dance had worked in general; - All participants experienced digital dance as safe and the only side effect mentioned was sore joints, which can be expected after physical exercise; - A compliance rate of 86% was measured at post-test. Twenty-one (91%) participants completed seven or more dance classes and two participants completed four classes; - 83% reported to be highly motivated to attend dance class; - None of the participants reported worse self-reported physical function, well-being, and cognitive function after 10 weeks of dancing; - 74% of the participants indicated that they perceived the artistic dimension to digital dance as an important element; - There were significant improvements at post-test in PDQ-39 summary index (p < 0.001). Subscales Mobility (p = 0.016) and Cognition (p = 0.005) were significantly improved at post-test. Post-hoc analysis for the mobility subscale (10 items) showed that no single item was driving the improvement. For the Cognition subscale (4 items), item 30 (daytime sleepiness) (p = 0.008) and item 33 (dreams and hallucination) (p = 0.008) showed significant improvements at post-test, but not item 31 (concentration) or item 32 (memory failures); - The depression subscale of the HADS questionnaire was significantly improved at post-test (p = 0.003); - There was no significant change between pre- and post-test for CIS, PRMQ or the two items from the Mental Fatigue Scale. |
|  |  |
| **Category: remote consultation** | |
| **Study** | **Other outcomes** |
| Anghelescu et al.  (2022) | - Participants reported feeling alone and unsupported during the pandemic; - Participants reported some uncertainty around the restrictions put in place and expressed concerns about how insurance works with increased dispensing fees; - Some participants reported a change in routine for obtaining their medication, including the new delivery service provided by pharmacy or shift in routine; - Some participants provided insightful recommendations including secure email and video methods to efficiently and remotely connect with physicians; - Many participants reported that the appointment’s motor examination was less useful to them and emphasized its importance for their neurologist; - Participants described the worsening of motor symptoms of PD since the pandemic started; - During the pandemic, PD patients expressed an increase in anxiety and stress, leaving some participants noticing more substantial changes as a result; - Many participants reported worsening of sleep during the pandemic, expressing increased difficulty falling asleep and a broken sleep during the night-time, and increased frequency of daytime naps; - Many participants reported abrupt changes in their lives with both cancellation and alteration of multiple routine social and physical activities they were engaged in; - Many participants reported their modified exercise routine during the pandemic now includes more walking outside during pleasant weather and adapting to virtual exercise classes either on their own via YouTube or following the Parkinson Association of Alberta exercise classes; - While some people live together with family and others alone, most of the participants have expressed missing their usual daily activities outside the home and visiting with friends and family taking a toll on their well-being; - While some participants have expressed feelings of isolation, others are searching or have found new activities to manage the boredom that comes from being isolated in their homes; - Participants frequently reported adhering to the physical distancing measures by eliminating friends and family regular indoor visitations; - Participants expressed ways to keep socializing amid the pandemic by adapting to use technology, phone or video calls, or visiting outdoors with physical distancing measures; - In terms of spousal relationships, participants shared a mix of reports, with some expressing increased appreciation for their spouse during the pandemic, while others felt the strain of living in close proximity; - Many participants reported abiding to public health measures in place and minimizing risks for getting infected; - Few patients reported having comorbidities and felt at even greater risk; - When asked about travel and socialization restrictions, most participants understood where the priority lies with the health experts in safety precautions even if they miss their usual activities; - Participants’ attitudes were negative around those who do not adhere to public health measures; - While many participants expressed minimal fears or concerns about COVID-19 because of the inability to do anything about the situation, others felt different and skeptical of the current situation and what the future will hold, including health, finances, and government decisions. |
| Dorsey et al.  (2010) | - Participants randomized to remote consultation completed 100% of their visit; - The nursing home participants completed 92% of their remote consultation visits as scheduled (One visit was missed because of illness but was completed two weeks later); - At the study’s conclusion, nine of ten remote consultation participants and all four usual care participants opted to receive their PD care via remote consultation; - Compared with usual care, those randomized to remote consultation experienced significant improvement in:   - Quality of life (3.4 point improvement vs. 10.3 point worsening on the PDQ-39; P = 0.04);   - Motor performance (0.3 point improvement vs. 6.5 point worsening on the motor UPDRS; P = 0.03). |
| Evans et al.  (2020) | - 75.4% of patient feedback forms were returned; - 89% of respondents agreed or strongly agreed that they were satisfied with the virtual clinic (VC); - 79% of appointments were successful (i.e., if the clinician felt that the outcome of the consultation was likely to have been the same as a face-to-face clinic); - Reasons that the consultation was unsuccessful included complex phase of disease, problems with the Parkinson’s KinetiGraph (PKG), needing a blood pressure reading and speech problems. - With regard to expenses, the VC design reduced some costs by eliminating the need for clinic premises, support staff and ambulance transport as well as reducing secretarial input; - VC also added value in terms of convenience, environmental benefits and in terms of infection control; - VC using PKG appeared to be more expensive than a normal clinic. |
| Mammen et al.  (2018) | - Feedback was received for a total of 149 cases, with between 1 and 4 visits per case; - Familiarity with the Internet was high (96% used the Internet or e-mail at home), and half had previously conducted a video call (54%); - Baseline satisfaction with usual care was high (83% satisfied or very satisfied); - Of 149 cases, 124 (83.2%) expressed positive perceptions of the virtual visits, nineteen expressed neutral perceptions (12.6%) and six (4%) expressed negative perceptions; - The mean score for general patient feedback (‘‘Participant virtual house call’’) suggested a relatively favorable opinion; - The overall mean score for patients’ dislike of virtual house calls was close to zero, which reflects neutrality in their comments rather than outright expression of dislike; - The mean score for general physician feedback (‘‘Doctor virtual house call’’) was lower compared with patients, perhaps reflecting a more tempered consideration of telemedicine; - Physicians’ comments on performing remote motor assessment were scored near zero, suggesting a balanced or neutral opinion. |
| Peacock et al.  (2020) | - 52% (95% CI 32–72) of participants endorsed a need to live in a larger urban center to get the best care for their PD; - 12% (95% CI 0–25) of participants disagreed with the sentiment of needing to live in a larger urban center to get the best care for their PD; - More than two-thirds of travelers (living >50 km from neurologist) and non-travelers (living ≤50 km from neurologist) report that they never cancel appointments (p = 0.20); - Travelers were more likely than non-travelers to avoid scheduling clinic appointments in the winter due to travel concerns (46% vs. 8%; p < 0.01); - Most participants (82%; 95% CI 64–100) expressed that they would be comfortable using videoconference technology for follow-up appointments with their neurologist; - The participants did not express concern about loss of information in videoconference visits compared to in-office appointments. |
| Quinn et al.  (2019) | - Significant changes were found in speech language pathology (SPL) across each assessment task. - Post hoc analyses identified significant improvements in SPL for sustained phonation pre-treatment (PRE) – post-treatment (POST) (p=0.012) and PRE- three months post-treatment (FU) (p=0.012); - Significant improvements were identified for SPL during reading both PRE-POST (p=0.017) and PRE-FU (p=0.012); - Significant improvement in SPL in the monologue task was found PRE-POST (p=0.036), however this was not maintained at FU (p=0.093); - No significant differences were identified POST-FU for any measure of SPL (sustained phonation p=0.779; reading p=0.674; monologue p=0.779); - No significant differences were identified across the time intervals for maximum fundamental frequency range or maximum phonation duration; - Statistical analysis failed to reveal any significant differences across assessment intervals for either psychosocial measure; - Participants indicated a high level of satisfaction with treatment effectiveness, acceptability of the telerehabilitation service delivery model and telerehabilitation service delivery overall; - A slight preference for face-to-face therapy was identified. |
| Stillerova et al.  (2016) | - There was no missing data for the self-report MDS-UPDRS subscales of motor and nonmotor symptoms and motor complications; - In the motor examination of each participant, between zero and seven items could not be completed, excluding the intentionally omitted items of rigidity and postural stability; - The median number of items missing for each participant was 2.0 (IQR 1.0–4.0); - Whilst rigidity and postural stability were expected to have a frequency of 11, due to intentional omission caused by the inability to conduct physical tests for the 11 participants over videoconference, rest tremor of the lower extremity had a similarly high missing item frequency of 10; - The biggest differences in scores between the face-to-face and the videoconference assessment for the four subscales of the MDS-UPDRS occurred in the motor examination, with the median difference between scores being 3.0 (IQR 1.5–9.0) out of possible 132 points for this subscale; - Within the motor examination, the biggest differences between face-to-face and videoconference scores were for participants 8, 9, and 11 who, respectively, had a difference of 18, 26, and 14 points out of possible 132 points; - The median travel time and travel distance savings when remotely assessing were 86.0 minutes (IQR 38.0–226.0) and 60.2 kilometers (IQR 24.3–278.0) per participant, respectively; - Participant feedback indicated that all participants rated their experience with using videoconferencing to monitor their PD symptoms as good or even excellent - The majority of participants identified with being “quite familiar” or “very familiar” using their respective videoconferencing software, whereas one participant identified with being “neither familiar nor unfamiliar” and two identified with being “very unfamiliar.” |
| Tarolli et al.  (2020) | - Around 92% of scheduled assessments were completed at the final remote visit; - The majority of incomplete assessments (80.6%) were patient reported outcomes that were meant to be completed on the tablet following the visit with the investigator; - Mean (standard deviation) duration between virtual vs in-person visits was 24.5 (15.5) days (range 11–151 days); - 84.2% of virtual visits occurred within 4 weeks of an in-person visit. - Agreement between in-person and remote assess was highest among the MDS-UPDRS II (ICC= 0.87), MDS-UPDRS IA (ICC= 0.82), and MDS-UPDRS IB (ICC= 0.81); - Observed agreement was high between the remote and in-person modified Rankin Scale (94%) and Schwab and England Activities of Daily Living (92%); - Moderate agreement was identified between in-person and remote performed motor assessments for both the UPDRS Part III (ICC= 0.51) and MDS-UPDRS part III (ICC= 0.43). |
| Wannheden et al.  (2020) | Part 1 (Workshops):   - None.   Part 2 (Questionnaire):   - The majority of the 37 questionnaire respondents (84%) perceived that they would benefit from an eHealth co-care service; - The lowest rate was found for the pre-visit forms, but this was still rated with a mode of 4 (Important); - For all other functionalities (self-tracking, graphical overview, self-care recommendations, asynchronous communication), the mode was 5 (Very important); - The 3 functionalities that were rated most important were the ability to send messages to HCPs (asynchronous communication), graphical overview, and self-tracking; - The ability to receive messages from HCPs got the fewest ratings as one of the 3 most important functionalities, followed by the pre-visit forms.   Suggestions of additional functionalities:   - Accumulated statistics; - Synchronization with new models of care; - Drug information and interaction alerts; - Reminders to take medication. |
| Willows et al.  (2020) | - The median total time for any kind of contact required during Telemedicine (TM)-assisted Levodopa-Carbidopa Intestinal Gel (LCIG) home titration in hours:minute was: 8 : 53 (4 : 11–14 : 38) for patients, 1 : 14 (0 : 29–1 : 52) for neurologists and 5 : 49 (2 : 46–10 : 03) for Duodopa nurse specialists (DNS); - The median total number of contacts per patient was 19 (12–37): 13 (68.4%) by TM, 1 (5.3%) by phone, and 5 (26.3%) home visits; - The median number or TM contacts between neurologist and patient without DNS were 2; - The DNS executed the majority of contacts using the TM, some by home visits and occasionally by phone; - When comparing to their own experience from previous titrations, 60% of the neurologists and 93% of the nurses considered the time spent for patient contact during TM-assisted titration to be less than during hospital titration; - The median total time for the LCIG titration period was 2.8 (2.0–13.8) days; - Median total free time without contact with any healthcare professional (HCP) and without individual pump handling was 2.7 (1.7–9.5) day; - The median percentage of patient total free time during TM-assisted LCIG titration period at home was 92% (69%–96%) - Of the 183 TM contacts, 7% were associated with a technical event, affecting 11 patients (73%); - The most common consequence of a technical event was replacement of TM session by phone call (18), reestablishment of connection (13), or delayed contact (9); - Only 2 of the scheduled contacts failed to take place as scheduled - The most common root-causes of the technical problems related to the digital link, such as failure to establish connection or low sound quality; - 13 technical problems with equipment, 3 non-intentional mishandlings of equipment, and 4 “other” events were reported; - No intentional misuse of TM equipment was recorded; - Overall, 86% of patients were very satisfied (6 or 7 on the 7-point Likert scale) using TM for LCIG home titration, whereas 93% of neurologists and 80% of DNS were very satisfied with how the TM method met their clinical needs; - 87% of patients were very satisfied with the communication with neurologist and DNS, and with the ease of using the equipment; - 93% said that they felt very secure being titrated at home using TM; - Most (93% and 87%) of the DNSs and neurologists, respectively, stated that they were very satisfied with the communication and 87% and 80% were very satisfied with the clinical assessment; - Yes/no questions revealed that the majority of patients (92.9%) and caregivers (88.9%) were able to do things at home that they could not have done if the patient was hospitalized; - After the experience of LCIG home titration, 92.9% of the patients and 100% of the caregivers preferred that the patient had not been hospitalized to start LCIG treatment; - The majority of the neurologists (73.3%) and DNSs (60.0%) felt no important limitations for clinical assessment by using TM, compared to hospital titration, since rigidity and balance could be estimated visually when the patient was moving in front of the camera; - Time for patient contact (real communication time) and time to set up for patient (booked communication time) were considered to be less by the neurologists (60.0% and 66.6%, respectively) and by DNS (93.3% and 60.0%, respectively), while time for other tasks between contacts were considered to be more for both neurologists (53.3%) and DNSs (93.3%); - Using TM home titration for start of LCIG infusion, 14 patients (93%) decided to undergo a PEG-J implantation and continue LCIG infusion, which is in the same range as previously reported - Improvement on the CGI-I was reported for all 14 patients that chose to continue with PEG-J surgery; 10 patients (71.4%) were “much improved” and 4 patients (28.6%) were “very much improved” at the end of titration. No serious adverse drug reactions and only one device complaint (nasojejunal tube occlusion) was reported. |
